# Physical Activity, Symptoms, Quality of Life and Exercise Program Preferences in People With Chronic Lymphocytic Leukaemia

**DOI:** 10.1002/jha2.70100

**Published:** 2025-07-10

**Authors:** Ellie E. Miles, Jennifer L. Nicol, Hatti Fowler, Amelia Roberts, Andrew T. Hulton, Caitlin Jeary, Renata Walewska, Sunil Iyengar, Erik D. Hanson, CLL Support Association, Andrea Sitlinger, David B. Bartlett

**Affiliations:** ^1^ Department of Haematology, School of Biosciences University of Surrey Guildford UK; ^2^ School of Human Movement and Nutrition Sciences The University of Queensland Brisbane Australia; ^3^ University Hospitals Dorset Bournemouth UK; ^4^ Haemato‐Oncology Unit Royal Marsden Hospital London UK; ^5^ Department of Exercise & Sport Science University of North Carolina Chapel Hill North Carolina USA; ^6^ Lineberger Cancer Center University of North Carolina at Chapel Hill Chapel Hill North Carolina USA; ^7^ CLL Support Association Chippenham UK; ^8^ Department of Hematologic Malignancies and Cellular Therapies Duke University School of Medicine Durham North Carolina USA; ^9^ Division of Medical Oncology Duke University School of Medicine Durham North Carolina USA

**Keywords:** cancer symptoms, chronic lymphocytic leukaemia, physical activity, quality of life, symptom management

## Abstract

**Background:**

Chronic lymphocytic leukaemia (CLL) has a heterogeneous lifelong course. While some patients never require treatment, most experience intermittent periods of active monitoring with other time points in active treatment. Most patients experience significant symptoms which negatively impact their quality of life (QoL). Although physical activity and exercise may help manage symptoms, it is unclear what disease‐related factors drive the physical inactivity observed in people with CLL.

**Methods:**

This study explored physical activity among people with CLL and assessed differences and relationships in treatment stage, symptoms, quality of life, and preferences for physical activity using an online questionnaire.

**Results:**

A total of 128 individuals with CLL [66 M/62F: mean age 67 ± 9.1 years (range 38–91 years)] completed the questionnaire. Those who are being/have been treated (*N* = 55) exhibited worse QoL (*p* = 0.018) and lower engagement in higher levels of physical activity (*p* = 0.045) when compared to their treatment naïve (*N* = 73) counterparts. Both groups had similar symptomology, with fatigue (∼77%) and insomnia (∼55%) being the most reported and associated with less likelihood of being physically active. Physically active participants reported better QoL (*p* = 0.020), physical functioning (*p* = 0.003) and role functioning (*p* = 0.020) as well as lower levels of fatigue (*p* = 0.036), pain (*p* = 0.017) and symptom burden (*p* = 0.026) compared to those who were insufficiently active. Although 79% of respondents wanted to engage in exercise programs for their CLL, 70% reported never receiving exercise guidance from their healthcare professionals.

**Conclusion:**

These findings highlight a significant need for targeted interventions to increase physical activity, likely improving QoL, in people with CLL. Furthermore, there is considerable interest from the CLL community in receiving exercise guidance; however, factors such as treatment status and symptomology should be considered when developing CLL‐specific exercise programs.

## 1 Introduction

Chronic lymphocytic leukaemia (CLL) is the most common adult blood cancer in the Western world, with approximately 3800 new cases diagnosed annually and over 20,000 prevalent cases in the United Kingdom [1, 2]. CLL primarily affects males (63% of cases) and is largely a disease of older adults, with an average age of diagnosis over 70 years old [3]. However, over the past decade, the incidence of CLL has increased by 25% among individuals aged 50–70 years, and is projected to keep rising as diagnostic tools are improved [1]. People with CLL experience a complex disease journey characterised by an increased risk for infections and secondary cancers, with high symptom burden and comorbidities that reduce their quality of life (QoL). Central to this are cancer‐associated and premature age‐associated reductions in physical function and physiologic reserve [4, 5]. Reduced functional performance is associated with poor survival, more infections and further reductions in QoL [4, 6]. Further compounding this complexity are the additional symptoms and reductions in QoL of those patients on active monitoring (i.e., dynamic monitoring, treatment naïve or watch and wait), which differs, in parts, from those receiving treatment [7, 8]. For those on active monitoring, the period between diagnosis and treatment is several years, and in some cases, treatment is never required and is associated with distress, anxiety, social isolation, and fatigue [7, 8]. Similarly, for those on treatment, there are treatment‐related symptoms (e.g., nausea, diarrhoea, bleeding, fatigue) that compound the years of reduced QoL experienced whilst awaiting treatment. Therefore, there is a growing need to understand the relationships between symptoms, QoL and physical function to determine whether interventions will reduce CLL‐specific symptoms and improve QoL [9].

Although increasing physical activity levels and exercise exposure are recommended for managing symptoms and improving QoL in several solid cancers [10], it is unclear whether this is generalisable to CLL. Compared to solid cancers, patients with haematologic malignancies have lower physical function, higher levels of fatigue and an increased risk of frailty [11]. Approximately 60%–70% of older adults with CLL are classified as either pre‐frail or frail, a striking contrast to the 15%–30% frailty observed in the general older population [12, 13]. Physical activity and exercise‐based interventions can often reduce frailty by improving physical function in both clinical and community settings [14]. Furthermore, in haematological malignancies, being more physically active is associated with a better QoL [15]. Recently, small pilot studies of exercise interventions in patients with CLL suggest that exercise training with behaviour change strategies to increase physical activity can increase physical fitness and physical function, lower symptom burden and improve overall QoL [16, 17, 18]. Despite the potential benefits of physical activity and enhanced physiological fitness for individuals with CLL, physical activity levels are 30%–40% lower than those of healthy individuals in the same age group [5]. It is unclear what factors drive this physical inactivity phenotype and whether there is an opportunity for healthcare professionals to intervene with lifestyle approaches. Therefore, it is crucial to identify and address factors influencing physical activity and exercise participation in this population while also gaining a deeper understanding of how physical activity impacts QoL in individuals with CLL.

This study aimed to investigate for the first time the relationships between physical activity, QoL, CLL symptoms, and comorbidities among individuals with CLL at different stages of treatment. In addition, we aimed to assess the beliefs and preferences related to physical activity and exercise advice and delivery to understand if there is an opportunity for healthcare professionals to promote exercise interventions.

## 2 Methods

### 2.1 Study Population

We recruited individuals diagnosed with CLL primarily through the CLL Support Association, UK. This patient‐led charity supports people with CLL or small lymphocytic leukaemia (SLL), families, and supporters. Recruitment was through word of mouth, online promotion, and clinic referrals. Information on participants’ diagnoses was self‐reported and collected through an initial screening questionnaire. People were eligible if they had a confirmed diagnosis of CLL or SLL of any treatment status, were aged 18 years or over, could understand the written English language. Eligible participants completed the consent form and gained access to the questionnaire via Qualtrics (Qualtrics LLC, USA). The University of Surrey Ethics Committee reviewed the protocol and gave a favourable ethical opinion (FHMS 21–22 261 EGA).

### 2.2 Survey Instrument Development

We collected data from this cross‐sectional observation study using an online version of a 183‐part questionnaire separated into eight sections (Figure S1). Briefly, the questionnaire captured: ***Section 1*** *(Items 1–8)*: Personal information. ***Section 2*** (*Items 9–33)*: Self‐reported health‐ and disease‐related characteristics [2]. ***Section 3*** *(Items 34–41)*: Self‐reported physical activity levels [19, 20, 21, 22, 23, 24, 25]. ***Section 4*** *(Items 42–75)*: Interest and advice on physical activity [24]. ***Section 5*** *(Items 76–121)*: Self‐reported quality of life (QoL) [26, 27, 28, 29]. ***Section 6*** *(Items 122–151)*: CLL Comorbidities Index [30, 31]. ***Section 7*** *(Items 152–176)*: Dietary analysis [32]. ***Section 8*** (*Items 177–183*): Additional but optional self‐reported health and disease characteristics [2].

### 2.3 Statistical Analysis

We conducted statistical analyses using SPSS v29.0 (IBM, USA). We report the characteristics of our study population using descriptive statistics such as means ± standard deviation (SD) or median and interquartile range (IQR), depending on data normality determined by the Kolmogorov–Smirnov test. For data presentation and interpretation, we also present frequencies/proportions. We used Pearson Chi‐square tests to assess categorical variables. Where data were normally distributed, we used *t*‐tests and ANOVAs to compare groups. Where data were not normally distributed, nonparametric Mann–Whitney (continuous variables), Kruskal–Wallis (more than 2 groups) and the Wilcox Signed Rank test for paired samples were used. When appropriate, effect sizes were assessed using the rank‐biserial correlation coefficient (*r*) and post hoc analysis was carried out using Dunn's pairwise test. Significance values reported from the Dunn's pairwise test have been adjusted by the Bonferroni correction for multiple tests.

Symptom intensities were described as: ‘not at all’ (1)/‘slightly’ (2)/’moderately’ (3)/‘significantly’ (4)/‘overwhelmingly’ (5) and collapsed into three categories: not at all (response 1), slightly (response 2), and moderately or greater (responses 3, 4, and 5). Preferences in exercise program responses were also collapsed into three categories to determine proportions. ‘strongly agree’ (1)/‘agree’ (2)/‘neutral’ (3)/‘disagree’ (4)/‘strongly disagree’ (5) was collapsed into agree (response 1 and 2), neutral (response 3), and disagree (response 4 and 5), while ‘very important’ (1)/‘important’ (2)/‘neutral’ (3)/‘unimportant’ (4)/‘very unimportant’ (5) was collapsed into important (response 1 and 2), neutral (response 3), and unimportant (response 4 and 5). Due to reduced numbers, we collapsed treatment status into two categories: Treatment‐Naïve (TN) and Treated (TRE).

Multivariable logistic regression analyses were conducted to assess the factors associated with being physically active. Potential predictors were independently modelled depending on their classification as either clinical/demographic (e.g., age, BMI, years with CLL, years treated for CLL, comorbidity index), symptoms or QoL indices. Physical activity exposure/levels were the dependent variable and dichotomised into either insufficiently active or moderate/highly active. We present adjusted odds ratios with 95% confidence intervals (95% CI). All statistical tests were two‐tailed, with an alpha level of ≤0.05 set for statistical significance, while an alpha level of ≤0.10 was considered a trend.

## 3 Results

### 3.1 Participant Characteristics

One hundred and twenty‐eight people with confirmed CLL/SLL [66 M/62F: mean age 67 ± 9.1 years (range 38–91 years)] with treatment naïve [*N* = 73 (57%)] or having received treatment [*N* = 55 (43%)] completed the questionnaires (Table 1). Briefly, both groups were a similar age (*p* = 0.145), while treated participants reported being diagnosed with CLL for around 2.8 years longer (*Z* = –3.737, *p* < 0.001, *r* = 0.334). As expected, more treated participants reported a higher Binet stage (*p* < 0.001) and a trend for 50% more comorbidities (*Z* = –1.839, *p* = 0.066, *r* = 0.163) than treatment naïve. The majority were white and had high education levels.

**TABLE 1:** Sociodemographic and clinical characteristics by treatment status.

	Treatment naïve (*N* = 73)	Treated (*N* = 55)	*p* value
**Demographics**			
Sex (male/female)	33/40	29/26	0.339
Age (years) [mean ± SD]	66.2 ± 10.3	68.2 ± 6.9	0.145
Age [*N* (%)]			
<40	1 (1.4)	0 (0.0)	
40–49	4 (5.6)	0 (0.0)	
50–59	13 (18.2)	8 (14.4)	
60–69	32 (44.8)	25 (45.0)	
70–79	17 (23.8)	21 (37.8)	
80–89	4 (5.6)	1 (1.8)	
≥90	2 (2.8)	0	
Ethnicity^a^ [*N* (%)]			0.013
White British	58 (79.5)	29 (52.7)	
White Other	15 (20.5)	21 (38.2)	
Asian	0	1 (1.9)	
Black	0	2 (3.6)	
BMI [mean ± SD]	25.15 ± 5.37	25.37 ± 3.95	0.798
Current smoker [*N* (%)]	7 (9.6)	2 (3.6)	0.221
**CLL characteristics**			
Binet stage [*N* (%)]			<0.001
Stage A	36 (49.3)	7 (12.7)	
Stage B	5 (6.8)	6 (10.9)	
Stage C	1 (1.4)	9 (16.4)	
Unknown	31 (42.5)	33 (60.0)	
Years with CLL [median (IQR)]	4.3 (1.9–8.5)	7.1 (4.8–12.6)	<0.001
Treatment duration [median (IQR)]	NA	1.5 (0.6–5.0)	
Treatments [*N* (%)]			
Currently receiving treatment		35 (63.6)	
BTK inhibitor monotherapy		15 (42.9)	
Venetoclax		2 (5.7)	
Venetoclax + rituximab		4 (11.4)	
Venetoclax + obinutuzumab		5 (14.3)	
Venetoclax + chlorambucil		1 (2.9)	
Venetoclax + ibrutinib		4 (11.4)	
Other		4 (11.4)	
Currently in first‐line therapy		26 (47.2)	
Total CIRS score [median (IQR)]	2.0 (0.0–4.0)	3.0 (1.0–6.0)	0.066
**Life characteristics**			
Education level [*N* (%)]			0.463
Secondary school to year 11	4 (5.5)	5 (9.1)	
Secondary school to sixth form/college	5 (6.8)	4 (7.3)	
Certificate or diploma	4 (5.5)	8 (14.5)	
University undergraduate degree	28 (38.4)	20 (36.4)	
University taught postgraduate degree	28 (38.4)	15 (27.3)	
University postgraduate research degree	3 (4.1)	3 (5.5)	
Health care provider [*N* (%)]			0.594
Private	12 (16.4)	11 (20.0)	
NHS	60 (82.2)	43 (78.2)	
Mode of transport to medical appointments^b^ [*N* (%)]			0.393
Private vehicle	43 (58.9)	30 (54.5)	
Public transport	3 (4.1)	4 (7.3)	
Combination of private and public	26 (35.6)	19 (34.5)	
By foot	0 (0.0)	2 (3.6)	
Other (e.g., taxi)	1 (1.4)	0 (0.0)	
Living arrangements [*N* (%)]			0.127
With partner/spouse	53 (72.6)	32 (58.2)	
With partner/spouse and children	4 (5.5)	1 (1.8)	
With partner/spouse and an independent adult	3 (4.1)	3 (5.5)	
With an independent adult and children	1 (1.4)	0 (0.0)	
With independent adult	2 (2.7)	7 (12.7)	
Alone	10 (13.7)	12 (21.8)	

BMI, body mass index; BTK, bruton tyrosine kinase; CIRS, Cumulative Illness Rating Scale; CLL, chronic lymphocytic leukaemia; NA, not applicable; NHS, National Health Service.

^a^ For ethnicity, multiple other options were available to select.

^b^ For transport methods, multiple other options were available.

### 3.2 Impact of Treatment Status on QoL

**FIGURE 1:**
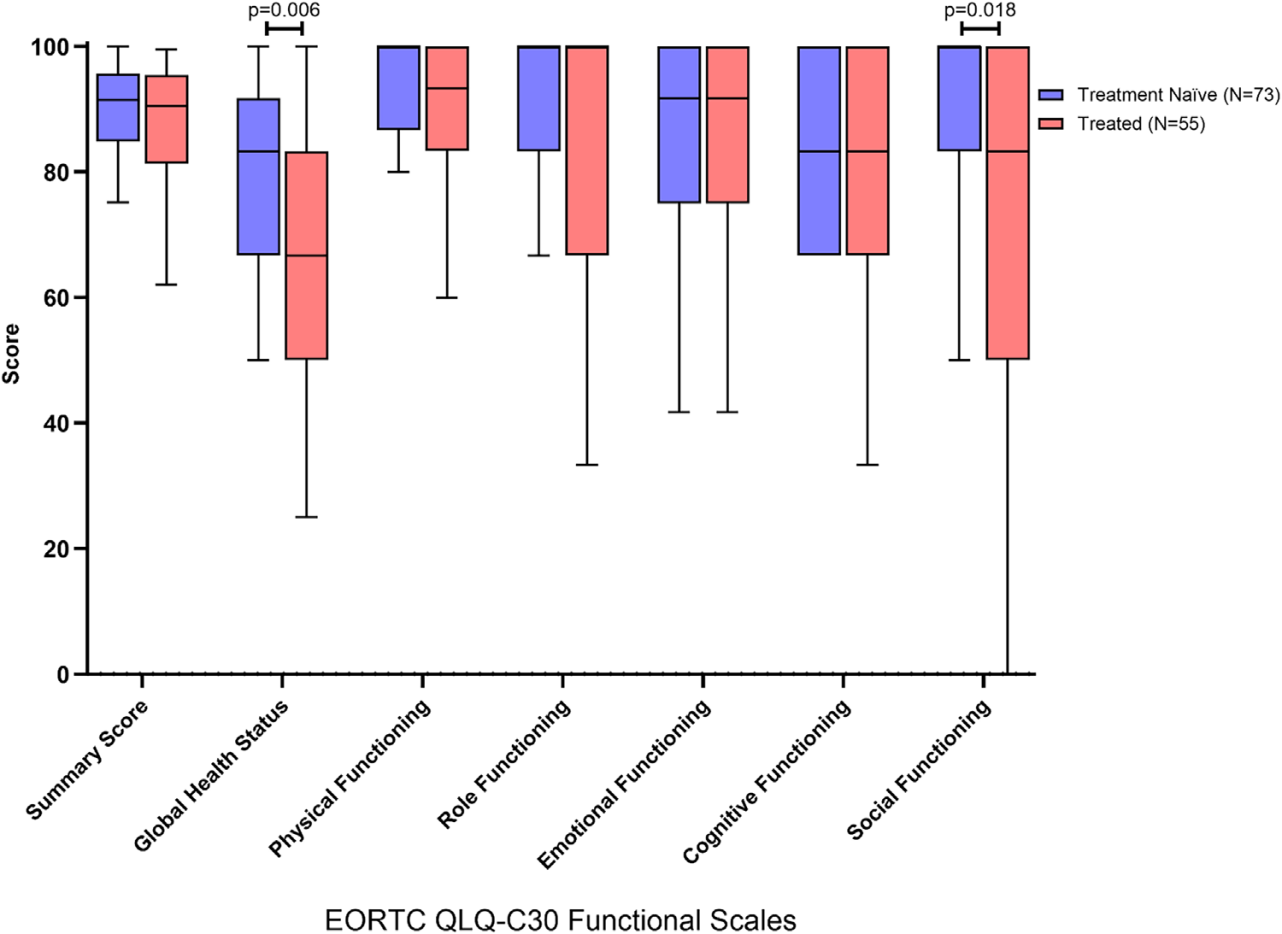
Comparison of global health status and functional scales of quality of life using EORTC QLQ‐30 v3 Questionnaire in treatment naïve CLL participants compared to treated CLL. All the scales range in scores from 0 to 100; a high score represents a higher (better) level of functioning. Boxplots are median and interquartile ranges with 10%–90% whiskers.

Figure 1 shows that treated participants had a lower QoL as measured by global health status (*Z* = –2.740, *p* = 0.006, *r* = –0.250) and lower social functioning (*Z* = –2.360, *p* = 0.018, *r* = –0.220). Additionally, treated participants showed a trend towards lower role functioning (*Z* = –1.710, *p* = 0.087, *r* = –0.160). No significant differences were found between groups on other functional scales (Table S2).

### 3.3 Impact of Treatment Status on Symptoms

**FIGURE 2:**
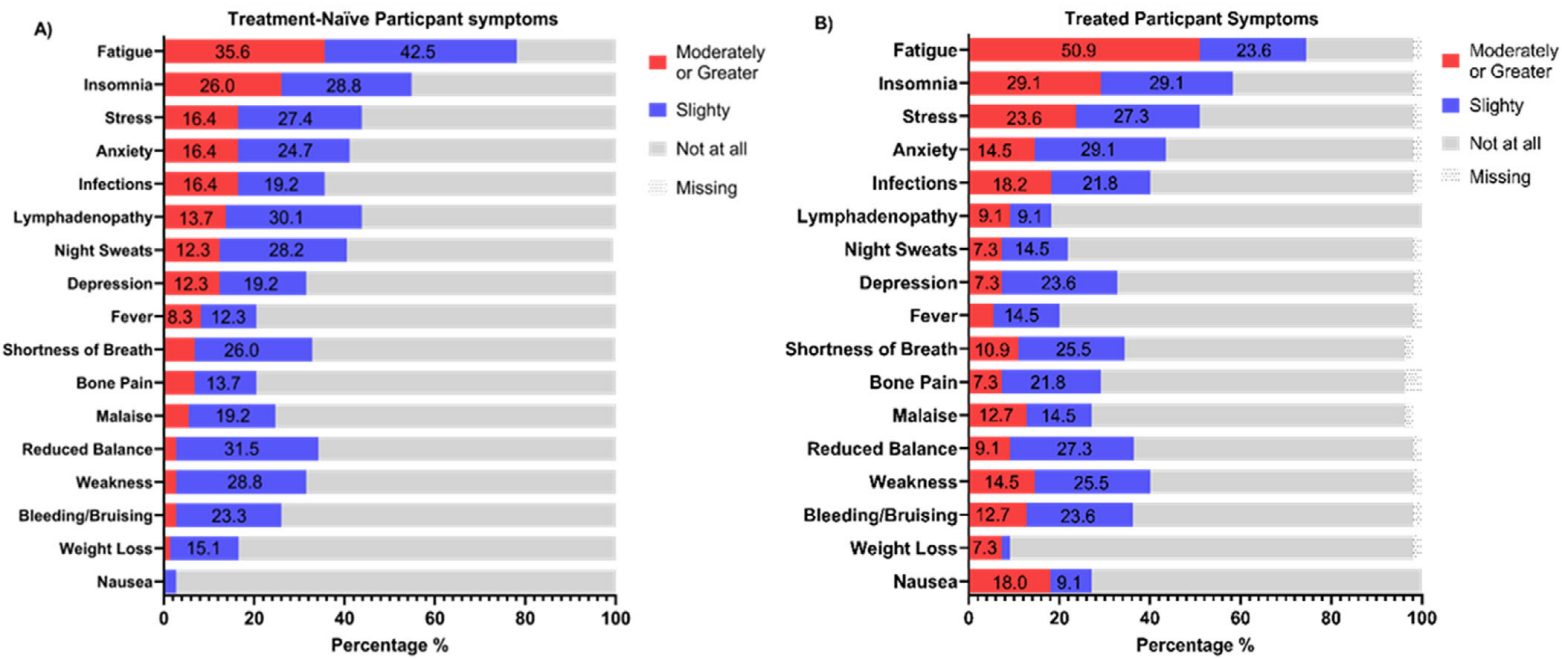
Intensity of symptoms over the previous month experienced by people with treatment naïve CLL (A: *N* = 77) and peoples who have been treated for CLL (B: *N* = 55).

The frequency and intensity of CLL symptoms experienced in the month before completing the survey are presented in Figure 2A (treatment naïve) and Figure 2B (treated). Most experienced at least one symptom at a moderate or greater intensity (65.3%), with 92.9% of individuals experiencing at least one symptom to a slight degree. Overall, fatigue (∼77% of participants) was the most reported symptom in both groups. Although both groups reported similar feelings of fatigue, approximately 15% more treated participants experience higher levels (*p* = 0.081). Similarly, more than 50% of participants in both groups reported insomnia, while 58.2% of treated participants reported stress. All other symptoms were reported in less than 50% of participants. When comparing other symptom profiles between groups, 25.6% more treatment naïve participants reported lymphadenopathy (*p* = 0.007) and twice as many treatment naïve participants reported unexpected weight loss (*p* = 0.012), while 10.2% more treated participants reported weakness (*p* = 0.044) (Table S3).

**FIGURE 3:**
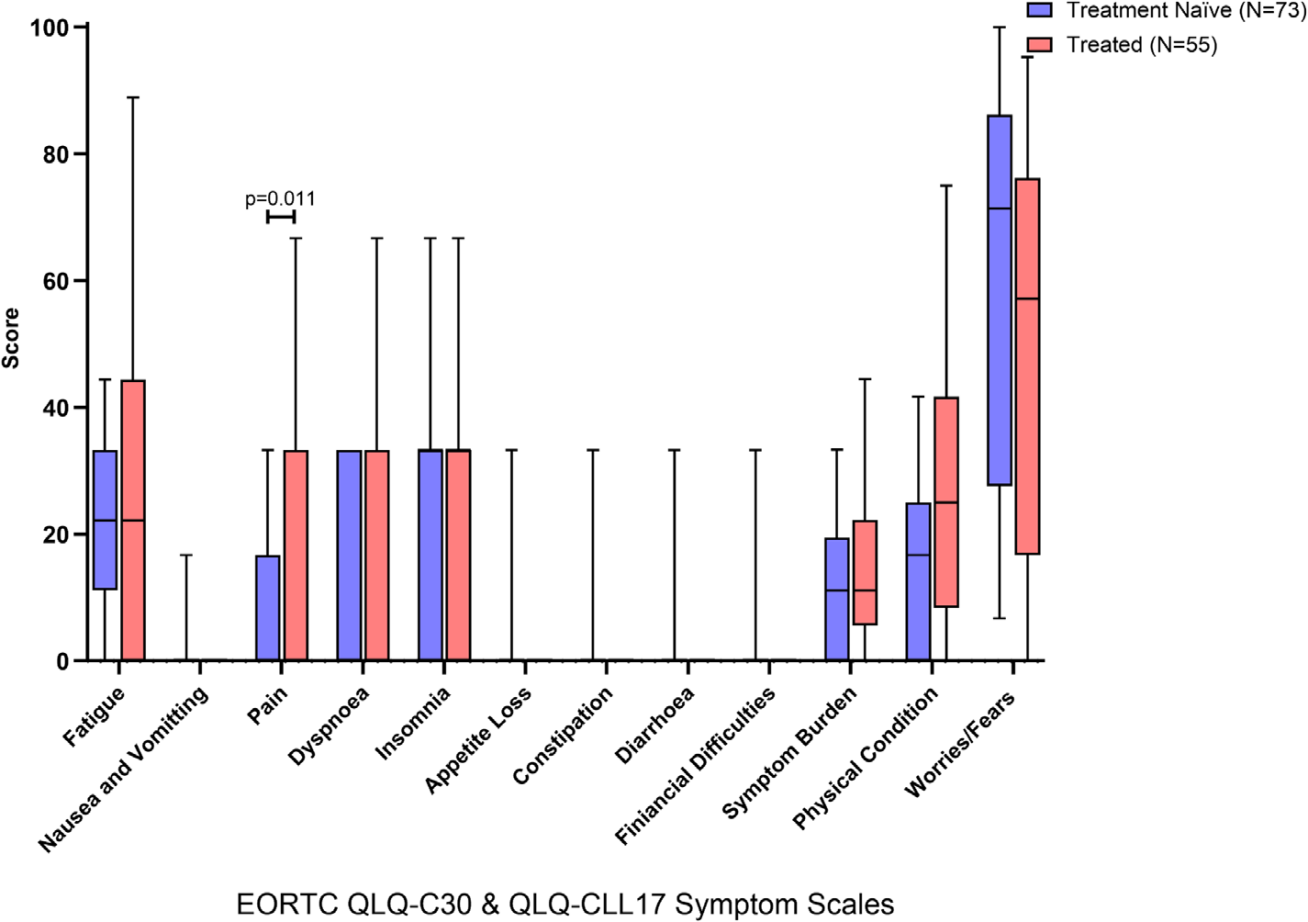
Comparison of symptom scales for quality of life in CLL participants using EORTC QLQ‐C30 v3 and EORTC QLQ‐CLL17 Questionnaire with treatment naïve compared to those who are treated. All the scales range in scores from 0 to 100; a high score represents a higher (worse) level of symptoms. Boxplots are median and interquartile ranges with 10%–90% whiskers.

When analysing symptom scales, treated participants reported higher levels of pain than treatment naïve (Figure 3: *Z* = –2.538, *p* = 0.011, *r* = –0.230). We observed trends towards a lower physical condition in treated participants (Figure 3: *Z* = –1.030, *p* = 0.075, *r* = –0.090) while treatment naïve exhibited trends towards higher worries and fears (Figure 3: *Z* = –1.680, *p* = 0.093, *r* = –0.150) (Table S4).

### 3.4 Impact of Treatment Status on Physical Activity Levels

**FIGURE 4:**
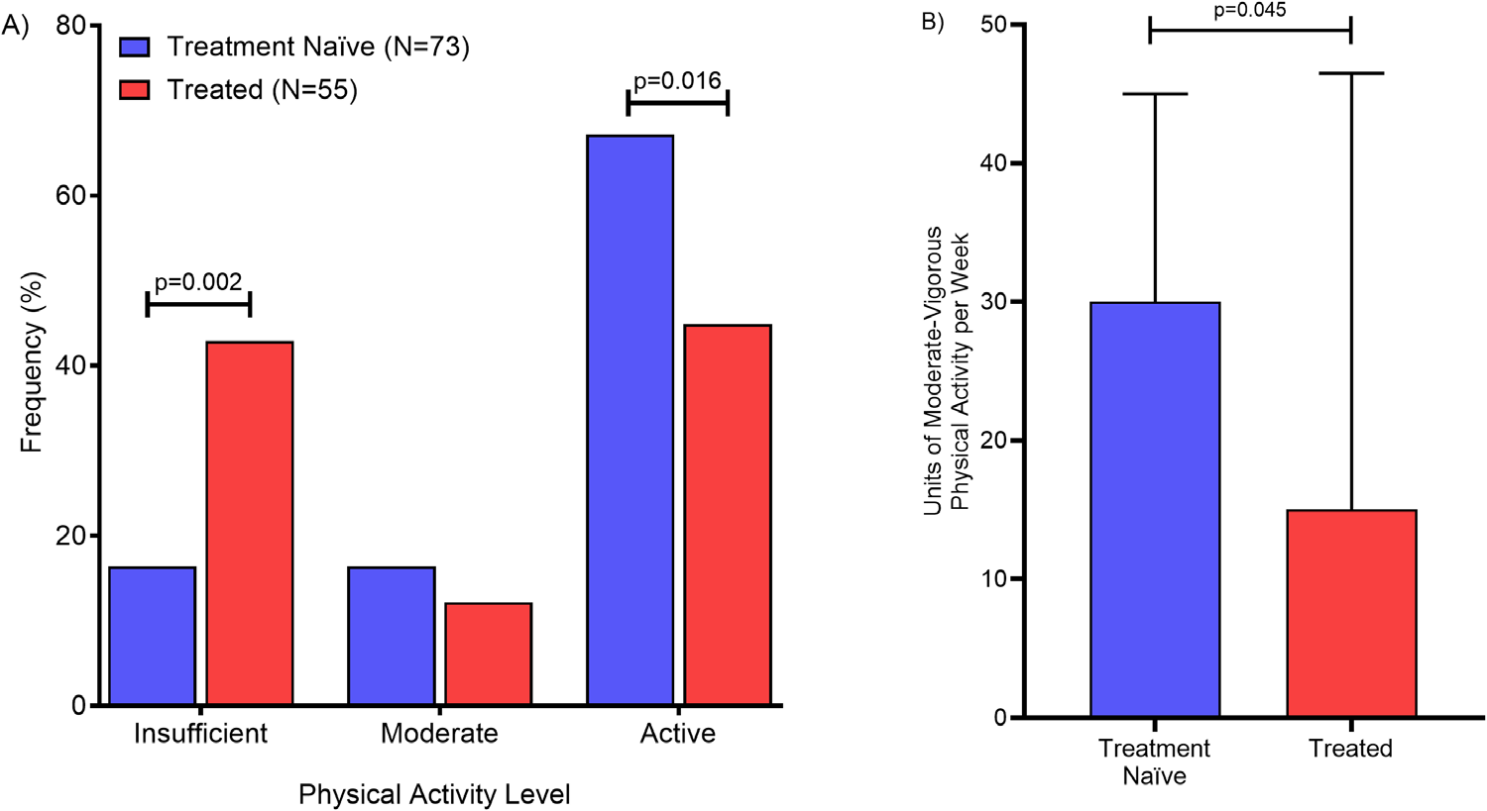
(A) Physical activity levels of CLL participants between treatment groups (treatment naïve vs. treated) using Godin Leisure Time Physical Activity Questionnaire. (B) Moderate‐vigorous physical activity units per week between treatment groups using the Godin Leisure Time Physical Activity Questionnaire. Values for physical activity levels per week are mean and SD.

The self‐reported physical activity levels of the cohort suggest that ∼70% of participants think they complete activities at moderate to hard intensities and amounts (Figure 4A). However, when analysing the number of minutes spent in moderate and vigorous activities, 24.6% of individuals meet physical activity guidelines of >150 min of moderate‐intensity activity and/or >75 min a week of vigorous‐intensity activity (Figure 4B). Physical activity levels between groups differed, with 26.5% more treated participants being insufficiently active (Figure 4A: *p* = 0.002) and 22.3% more treatment naïve participants being active (Figure 4A: *p* = 0.016). Additionally, treatment naïve individuals completed around twice as many moderate and vigorous minutes per week [treatment naïve: median = 30.0 (IQR: 20.0–43.0) vs. treated: median = 15.0 (IQR: 5.0–46.5), *p* = 0.045, Figure 4B]. There were no significant differences between those who met physical activity guidelines before diagnosis (29.9%) and those currently meeting (24.6%) physical activity guidelines (Tables S5 and S6).

### 3.5 Impact of Physical Activity Levels on QoL and Comorbidities

**FIGURE 5:**
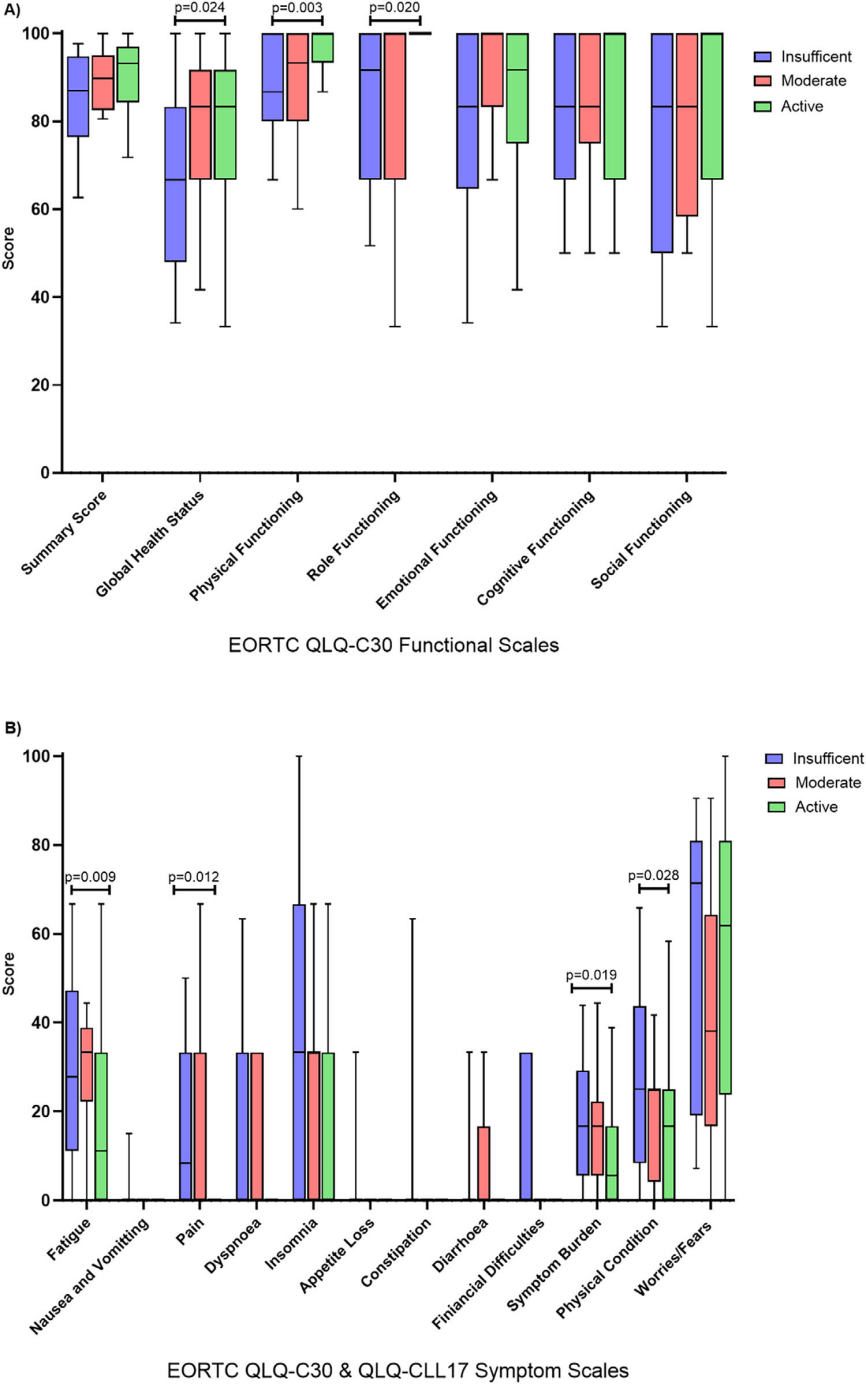
(A) Comparison of global health status and functional scales of quality of life in CLL participants with different physical activity levels categorised based on the Godin Leisure Time Physical Activity Questionnaire. (B) Comparison of symptom scales in quality of life in CLL participants with different physical activity Levels based on the Godin Leisure Time Physical Activity Questionnaire. All the scales range from 0 to 100; a high score represents a higher (better) level of functioning or a higher (worse) level of symptoms. Questionnaire. All the scales range from 0 to 100; a high score represents a higher (better) level of functioning or a higher (worse) level of symptoms. Boxplots are median and interquartile ranges with 10%–90% whiskers.

CLL participants with insufficient physical activity levels exhibit lower global health status and functional scales than those who are sufficiently active (Figure 5A). Specifically, physically inactive participants have the lowest global health status, a key indicator of QoL (*p* = 0.024) and the lowest role functioning (*p* = 0.020) compared to their more active counterparts. Moreover, participants engaging in insufficient (*p* = 0.003) or moderate (*p* = 0.042) levels experienced lower physical functioning than the most active (Table S7).

CLL participants with insufficient physical activity levels reported significantly higher symptom severity than sufficiently active participants in several categories (Figure 5B). Specifically, higher levels of fatigue (*p* = 0.036), pain (*p* = 0.017), overall symptom burden (*p* = 0.026), and a decline in physical condition (*p* = 0.023) compared to those classified as active. However, there were no significant differences in these symptoms between insufficiently and moderately active participants, nor between moderately‐ and highly‐ active participants (Table S8).

### 3.6 Associations Between Symptoms, QoL and Being More Physically Active

Multivariable linear regression analysis was used to determine the relationships between clinical factors, symptoms, QoL and whether people met physical activity guidelines (Table S1). CLL treatment [OR 0.262; 95% CI (0.111, 0.618)], fatigue [OR 0.979; 95% CI (0.960, 0.998), *p* = 0.033], dyspnoea [OR 0.978; 95% CI 0.958, 0.999, *p* = 0.036], insomnia [OR 0.983; 95% CI (0.969, 0.997), *p* = 0.017], physical condition [OR 0.967; 95% CI (0.945, 0.990), *p* = 0.005], and symptom burden [OR 0.960; 95% CI (0.929, 0.992), *p* = 0.014] were associated with a reduced likelihood of meeting physical activity guidelines. Additionally, a trend was observed for pain [OR 0.981; 95% CI (0.962, 1.001), *p* = 0.066] being associated with a reduced likelihood. Furthermore, QoL as measured by global health status [OR 1.029; 95% CI (1.007, 1.051), *p* = 0.008] and physical functioning [OR 1.059; 95% CI (1.015, 1.105), *p* = 0.008] were associated with the likelihood of meeting physical activity guidelines while role [OR 1.015; 95% CI (0.997, 1.034), *p* = 0.099] and emotional [OR 1.012; 95% CI (0.999, 1.037), *p* = 0.069] functioning and the Summary Score [OR: 1.038; 95% CI (0.996, 1.081), *p* = 0.074] trended towards the likelihood of meeting guidelines.

Since individual factors associated with physical activity were strongly related to each other, many were not independently predictive in a multivariate stepwise mode. Both treatment status (OR 0.221; 95% CI 0.072, 0.0673, *p* = 0.008) and physical condition [OR 0.930; 95% CI (0.875, 0.988), *p* = 0.019] were independently associated with physical activity.

Given that physical activity levels fluctuate, and some people hover between meeting and achieving recommendations, we determined the relationships between symptoms and the amount of physical activity. Symptom burden [*β* = –0.214 (95% CI: –0.833, –0.083), *p* = 0.017] independently explained 4.6% of the variation in physical activity scores [*F*(1, 122) = 5.85, *p* = 0.017]. As symptom burden is a construct of several different symptoms, we ran a second multivariable analysis to determine which symptoms were associated with symptom burden. We found that fatigue [*β* = 0.413 (95% CI: 0.155, 0.337), *p* < 0.001], insomnia [*β* = 0.270 (95% CI: 0.061, 0.179), *p* < 0.001] and pain [*β* = 0.271 (95% CI: 0.078, 0.265), *p* < 0.001] independently explained 58.2% of the variation in symptom burden [*F*(13, 114) = 52.84, *p* < 0.001].

### 3.7 Exercise Programme Preferences

**FIGURE 6:**
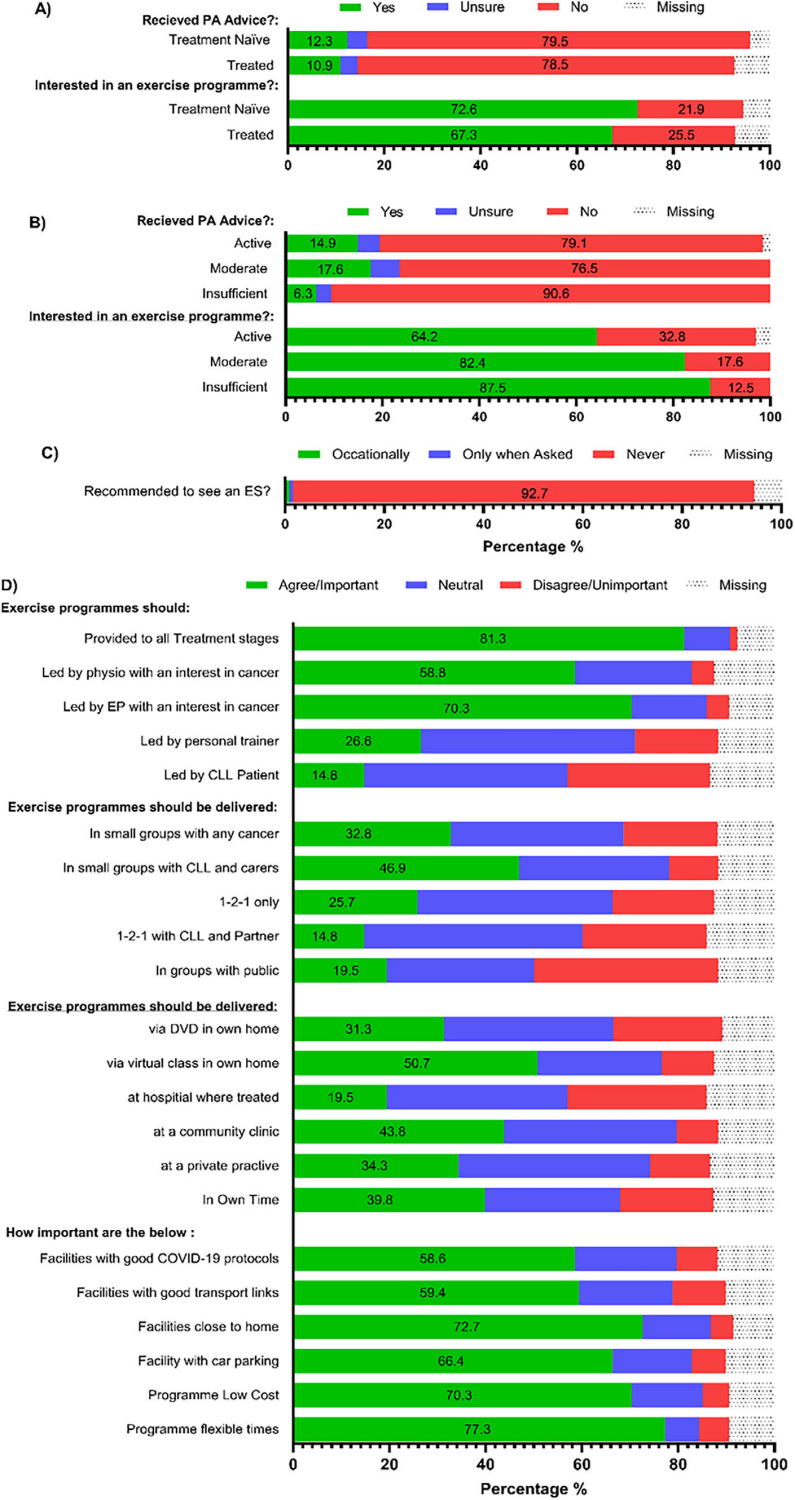
(A) Reported physical activity advice received from health care professionals in treatment naïve CLL participants compared to treated CLL. (B) Reported physical activity advice received from health care professionals in CLL participants with different physical activity levels categorised based on the Godin Leisure Time Physical Activity Questionnaire. (C) Respondents interested in an exercise programme. (D) Respondent's preferences for treatment stage, location, composition, and delivery of exercise programme. PA, physical activity; ES, exercise specialist; EP, exercise physiologist.

Most participants (70.3%) reported never receiving guidance regarding exercise or maintaining physical activity following a CLL diagnosis from healthcare professionals (Figure 6A). Despite this lack of guidance, 78.9% of respondents expressed interest in participating in an exercise program (Figure 6B). Among those who were not interested, 63.3% believed they were already sufficiently physically active and, therefore, not interested in a programme. Additional reasons for lack of interest included the perception that the program needed to be explicitly tailored to CLL (10%), a preference for exercising alone (10%) and concerns about travel distance (6.6%).

Participants strongly preferred supervised exercise sessions led by professionals specialising in cancer care (Figure 6C and D). Specifically, most favoured exercise physiologists (70.3%) and physiotherapists (58.6%) with cancer expertise over personal trainers (26.6%) and other people with CLL (14.8%). Additionally, respondents favoured programs with a social component. These included those with other cancer (68.7%) or with CLL (78.2%) participants, and not with the general public (19.5%). There were clear preferences for exercise programs that offered flexible scheduling (77.3%), were low‐cost (70.3%), had consistently available parking (66.4%), were located close to home (72.7%), had good transport links (59.4%), and adhered to robust COVID‐19 safety protocols (58.6%). There were preferences for virtual programs (76.3%), community‐based clinics (79.7%), private practices run by physiologists/physios (74.2%) but not at their hospitals (19.5%).

## 4 Discussion

The relationships between CLL and a reduced QoL are well documented; however, there remains limited information about how potential lifestyle approaches can offset or improve QoL. We report the findings from a UK‐based CLL survey that evaluates QoL, symptoms, treatment status and their relationships with physical activity levels. As expected, treatment of CLL was associated with worse QoL indices, while fatigue, insomnia and stress/worries/fears were the most commonly reported symptoms in both treatment groups. Although our cohort self‐reported a relatively high engagement in moderate‐ to vigorous‐intensity physical activity, less than one‐quarter met the recommended amounts of physical activity per week. People who had not received treatment for CLL engaged in more moderate‐ to vigorous‐intensity physical activity per week and reported a better QoL but had similar symptomology to those who had received treatment. Physically active individuals had lower fatigue, pain, and symptom burdens and a better QoL, as measured by global health status. In multivariate analyses, we found that treatment status and physical condition were independently associated with completing physical activity guidelines. Furthermore, symptom burden, a construct of fatigue, insomnia and pain, was independently associated with the amount of physical activity completed. Our data suggests that engagement with physical activity could help improve QoL by reducing symptoms, which could lower fatigue. However, we highlight a lack of advice about physical activity and exercise participation even though most people (especially those who are inactive) would like specialised physical activity advice and programs from their healthcare professionals.

### 4.1 Treatment Status, QoL and Physical Activity (QoL vs. Symptoms)

Our findings indicate that people with CLL who are treatment naïve experienced a better QoL. Specifically, they had better global health status, social functioning and trends towards better role functioning, similar to previous studies in cohorts from other countries [6, 8, 33, 34]. Additionally, both treatment groups reported low cognitive functioning scores. Although we did not assess healthy age‐matched controls, we can assume and compare from previous studies that even our treatment naïve group scores are lower than the general population. Several disease‐related factors have been suggested to contribute to this impairment in QoL [6, 8, 33, 34]. Unlike other low‐grade lymphomas, CLL is associated with increased opportunistic infections caught primarily through exposure during social and family situations. Such infections are associated with significantly higher chances of hospitalisation with patients who are on immune‐suppressing targeted therapies at even higher risks [35]. As such, the treated group with lower social functioning and a trend towards lower role functioning are likely due to such risks associated with CLL and exposure to environmental factors. This is consistent with literature indicating that cancer treatments can disrupt social relationships and reduce participation in social activities, leading to feelings of isolation, particularly among haematological malignancies [36]. Interestingly, while the treated group reported poorer global health status and social function, both groups expressed high levels of worry related to health and functioning, suggesting that psychological distress is a common experience among people with CLL regardless of treatment status [8, 34]. In the treatment naïve group, this anxiety could stem from the uncertainty surrounding disease progression, while the treated group likely worry about treatment‐related side effects, recurrence and risk of transformation.

Considering this, we show that CLL treatment is associated with completing more light and less hard physical activity and less likelihood of achieving physical activity guidelines. Additionally, being more physically active was associated with a better QoL. Specifically, higher physical activity levels were associated with higher global health status and better physical and role functioning. Our findings support those of others, where physical activity and exercise training can improve several aspects of QoL in solid malignancies, lymphoma and CLL [10, 12, 16, 18]. Physical and role functioning are obvious targets of physical activity and exercise, whereby active people are more inclined to complete basic, instrumental and everyday living tasks. Physical activity in older age is usually associated with more social interactions and may teach people with CLL how to integrate their disease with society. Although not significant, we observed that more active people also had higher cognitive and social functioning scores, similar to others [37]. As such, healthcare professionals may underappreciate the risks and benefits of physical activity. When patients are put on active monitoring, they feel that ‘nothing is being done’ and those on treatment are advised to avoid areas of infection risk [38]. As such, patients can lose the sense of control over their disease, which undoubtedly reduces their QoL. It is clear that being physically active and engaging in regular exercise is associated with enhanced immune function and reduced risks of opportunistic infections and cancer occurrence [39]. In CLL, only 12 weeks of exercise training has the potential to improve immune function and QoL [16, 40]. Although it is not surprising that physical activity and exercise improves the QoL, CLL also causes several important disease‐related symptoms that may further prevent physical activity engagement.

### 4.2 Treatment Status, Symptoms and Physical Activity

In both our groups, fatigue, insomnia, stress, anxiety and infections were highly reported. Like others who have analysed symptoms of CLL, fatigue was the most reported symptom, and those who were treated for CLL reported more severe fatigue [34]. However, the more active people were, the lower their fatigue levels were. Fatigue levels in several cancers have been shown to improve following exercise and physical activity interventions, albeit the mechanisms remain unknown [41]. Our regression analyses show that higher CLL symptom burden, primarily a construct of fatigue, insomnia and pain, was associated with less likelihood of being physically active.

Additionally, symptoms such as poor physical condition were independently associated with not completing physical activity guidelines. Taken together, our data suggests that targeting CLL symptoms has the potential to reduce fatigue and improve their QoL. This is consistent with studies that target the constructs of ageing conditions such as frailty [14], which is also highly prevalent in CLL. By targeting specific symptoms rather than generalised exercise/physical activity, exercise appears as if it can be utilised as a ‘medicinal’ intervention that not only treats symptoms but alleviates factors reducing QoL [18, 42]. As such, given that the more active participants reported lower symptom burden and better physical condition, physical activity likely plays a prominent role in CLL symptom management. In other cancers, particularly pre‐surgical cancers, multidisciplinary approaches, including exercise, diet and well‐being, are utilised to enhance physical function and limit cancer side effects [43]. That said, fatigue is clearly associated with the pathophysiology of CLL, as evidenced by high fatigue levels in treatment naïve patients. Therefore, although physical activity‐based approaches could reduce fatigue, in some cases (i.e., higher disease burden), the disease pathology may prevent improvements, and maintenance would be considered a positive response. However, such approaches are not offered for people with CLL but are becoming more common in stem cell transplant, showing improved physical condition and fitness [40]. Therefore, to investigate whether people with CLL would want such approaches, we asked them about their experiences and preferences.

### 4.3 Physical Activity Preferences

Similar to patients with multiple myeloma, nearly 80% of our participants suggested they would like to participate in exercise and physical activity programmes [24]. However, with 70% having never received guidance from their healthcare professionals and 93% never being recommended to see an exercise specialist, this is a complex conundrum. Not only are most people with CLL older, but they are also immunocompromised, which increases anxiety about safety. Indeed, safety appears to be a primary reason healthcare professionals do not engage in physical activity discussions and referrals [44]. Considering that exercise has been shown to be safe, feasible and provide powerful health benefits in CLL and other haematologic malignancies, the lack of guidance is concerning [16, 17, 18, 45]. However, given that the majority of our participants would not want to exercise at the hospital they are treated at and the lack of facilities in many hospitals, it is understandable that healthcare professionals do not know how to engage patients.

Indeed, most people would want to exercise in a virtual class at home or at a community clinic, not in public groups. Critically, cost, flexibility, and distance from home were most desired if sessions were outside of their home. As such, participants agreed more with exercise programs supervised by cancer care specialists, such as exercise physiologists and physiotherapists. Only a small percentage were interested in programs led by fellow CLL participants, and just 26.6% supported guidance from personal trainers. This suggests that participants place a high value on clinical expertise rather than on peer‐led or general fitness instruction, possibly due to the complex nature of their condition, just not in a hospital environment. Taken together, our survey indicates a desire for patients with CLL to be provided with exercise programs at all stages of their disease and to be included as part of clinically focused care. At present, there are no plans for patients with CLL to receive prehabilitation or rehabilitation programs on the NHS; as far as we can determine, no countries provide these services.

### 4.4 Strengths and Limitations

Strengths of our survey include a good representation of people with CLL in the United Kingdom, including treatment conditions, all disease stages, ages and respondents from all education levels. There is always a tendency for younger people who engage in physical activity to respond more to these surveys. However, this was not apparent in our survey. Other strengths included using validated questionnaires that distinguish between active and inactive older populations and have been used to assess physical activity levels in patients with cancer [20, 21, 23]. Secondly, recruitment through a CLL charity database is a strength. The database consists of almost 10% of the total number of people with CLL in the United Kingdom, providing enough power to accurately represent the CLL population. That said, a potential limitation of our study is that our participants were highly educated. Although similar numbers to the general population hold an undergraduate degree, almost 40% of our group hold postgraduate qualifications. While lower‐educated people can be less physically active and have more comorbidities, this was not evident in our cohort. That said, including more from this demographic would likely only have strengthened our results; however, future work should focus on including people from different demographic backgrounds to confirm this. Other limitations include relying on self‐reported questionnaires to evaluate physical activity levels, which introduces vulnerability to recall and social desirability biases, especially when relying on retrospective assessments before diagnosis. Further, it is difficult to rule out reverse causation using such questionnaires. This could have led to over‐ or underestimating levels. Using an online survey to collect data poses a limitation, particularly in older individuals and may have contributed to the skewing of education levels. This population may have limited access to or comfort with digital technology, potentially excluding less digitally literate participants and skewing the sample toward those more familiar with online platforms. Finally, although we asked people about their blood counts, including the prevalence of anaemia, less than 10% of participants knew their values or responded to the questions. Given that we have shown previous relations between physical dysfunction and blood markers, this could have added more depth to our understanding.

### 4.5 Conclusion

Our findings highlight the importance of and desire for targeted interventions to increase physical activity to potentially improve QoL by reducing symptoms, such as fatigue, in individuals with CLL. Our findings suggest that CLL care would likely improve by including exercise advice and prescriptions from healthcare professionals. Currently, most physical activity and exercise approaches employ a ‘cookie‐cutter’ approach and try to fit everyone into one model. We show that several factors, including treatment status and symptomology, should be considered when creating CLL‐specific programs. Future work is needed to understand the optimal amounts and timing of interventions and the degree of multimodality (e.g., nutrition, well‐being and exercise) required while also exploring patient preferences for exercise programmes further to improve accessibility and adherence. Moreover, further research is warranted to clarify the bidirectional relationship between fatigue and physical activity, which could inform more effective, personalised interventions.

## Conflicts of Interest

The authors declare no conflicts of interest.

## Supporting information




**Supporting File 1**: jha270100‐sup‐0001‐SuppMat.docx.

## Data Availability

The data that support the findings of this study are available from the corresponding author upon reasonable request.
