# Supplementary material for: Physical Activity, Symptoms, Quality of Life and Exercise Program Preferences in People With Chronic Lymphocytic Leukaemia
Source: EJHaem. 2025 Jul 10;6(4):e70100. doi: 10.1002/jha2.70100 (PMC12244265; doi:10.1002/jha2.70100)
Supplement: Supplementary file 1 — Supporting File 1: jha270100‐sup‐0001‐SuppMat.docx. [file JHA2-6-e70100-s001.docx]

**Supplementary Table 1**. Univariable and multivariable logistic regression models to assess relationships with CLL symptoms and QoL factors and being physically active

|  | Univariate | | Multivariate | |
| --- | --- | --- | --- | --- |
|  | OR (95% CI) | P value | OR (95% CI) | P value |
| **Clinical** |  |  |  |  |
| Age | 1.000 (0.957, 1.045) | 0.989 |  |  |
| Sex | 1.247 (0.551, 2.818) | 0.596 |  |  |
| BMI | 1.013 (0.907, 1.131) | 0.821 |  |  |
| CLL Years | 0.961 (0.889, 1.038) | 0.311 |  |  |
| Treatment Status | 0.262 (0.111, 0.618) | **0.002** | 0.221 (0.072, 0.673) | **0.008** |
| Treatment Type | 1.147 (0.883, 1.490) | 0.304 |  |  |
| Comorbidity Index | 0.936 (0.817, 1.073) | 0.342 |  |  |
| **CLL Symptoms** |  |  |  |  |
| Fatigue | 0.979 (0.960, 0.998) | **0.033** |  |  |
| Dyspnoea | 0.978 (0.958, 0.999) | **0.036** |  |  |
| Insomnia | 0.983 (0.969, 0.997) | **0.017** |  |  |
| Physical Condition | 0.967 (0.945, 0.990) | **0.005** | 0.930 (0.875, 0.988) | **0.019** |
| Symptom Burden | 0.960 (0.929, 0.992) | **0.014** |  |  |
| Pain | 0.981 (0.962, 1.001) | 0.066 |  |  |
| Nausea | 1.022 (0.951, 1.098) | 0.556 |  |  |
| Loss of Appetite | 0.989 (0.962, 1.016) | 0.414 |  |  |
| Constipation | 1.000 (0.982, 1.017) | 0.956 |  |  |
| Diarrhoea | 0.996 (0.974, 1.020) | 0.766 |  |  |
| Financial Difficulties | 0.985 (0.967, 1.004) | 0.127 |  |  |
| Worries/Fears | 0.997 (0.984, 1.010) | 0.621 |  |  |
| **QoL Factors** |  |  |  |  |
| Global Health Status | 1.029 (1.007, 1.051) | **0.008** |  |  |
| Physical Functioning | 1.059 (1.015, 1.105) | **0.008** |  |  |
| Role Functioning | 1.015 (0.997, 1.034) | 0.099 |  |  |
| Emotional Functioning | 1.018 (0.999, 1.037) | 0.069 |  |  |
| Cognitive Functioning | 1.011 (0.989, 1.032) | 0.325 |  |  |
| Social Functioning | 1.012 (0.997, 1.028) | 0.124 |  |  |
| Summary Score | 1.038 (0.996, 1.081) | 0.074 |  |  |

The dependent variable is the Godin dichotomous classification of physical activity levels. All significant univariate variables were considered as candidate predictors in the multivariable model.

**Supplementary Table 2.** Comparison of global health status and functional scales of quality of life using EORTC QLQ-30 v3 Questionnaire in treatment-naïve CLL participants compared to treated CLL. All the scales range in scores from 0 to 100; a high score represents a higher (better) level of functioning.

|  | **Treatment Naïve** | **Treated** | **P value** |
| --- | --- | --- | --- |
|  | **Median (IQR)** | **Median (IQR)** |  |
| Summary Score | 86.7 (81.3-95.5) | 91.4 (84.9-95.7) | 0.282 |
| Global Health Status | 83.3 (66.7-100.0) | 66.7 (50.0-83.3) | **0.006** |
| Physical Functioning | 100.0 (86.7-100.0) | 93.3 (83.4-100.0) | 0.484 |
| Role Functioning | 100.0 (83.8-100.0) | 100.0 (66.7-100.0) | 0.087 |
| Emotional Functioning | 91.7 (75.0-100.0) | 91.7 (75.0-100.0) | 0.671 |
| Cognitive Functioning | 83.3 (66.7-83.3) | 83.3 (66.7-100.0) | 0.719 |
| Social Functioning | 100.0 (83.3-100.0) | 83.3 (50.0-100.0) | **0.018** |

**Supplementary Table 3.** Comparison of differences in the intensity of symptoms experienced over the previous month between people with treatment naïve CLL (N=77) and people who have been treated for CLL (N=55).

|  |  | **Treatment Naïve**  **(*N* = 73)** | **Treated**  **(*N* = 54)** | **P value** |
| --- | --- | --- | --- | --- |
| **Symptom** | **Symptom Intensity** | **N (%)** | **N (%)** |  |
| Fatigue |  |  |  | 0.081 |
|  | Not at All | 16 (21.9 | 13 (24.1) |  |
|  | Slightly | 31 (42.5) | 13 (24.1) |  |
|  | Moderately and above | 26 (35.6) | 28 (51.9) |  |
| Insomnia |  |  |  | 0.862 |
|  | Not at All | 33 (45.2) | 22 (40.7) |  |
|  | Slightly | 21 (28.8) | 16 (29.6) |  |
|  | Moderately and above | 19 (26.0) | 16 (29.6) |  |
| Stress |  |  |  | 0.522 |
|  | Not at All | 41 (56.2) | 26 (48.1) |  |
|  | Slightly | 20 (27.4) | 15 (27.8) |  |
|  | Moderately and above | 12 (16.4) | 13 (24.1) |  |
| Anxiety |  |  |  | 0.819 |
|  | Not at All | 43 (58.9) | 30 (55.6) |  |
|  | Slightly | 18 (24.7) | 16 (29.6) |  |
|  | Moderately and above | 12 (16.4) | 8 (14.8) |  |
| Infections |  |  |  | 0.840 |
|  | Not at All | 47 (64.4) | 32 (59.3) |  |
|  | Slightly | 14 (19.2) | 12 (22.2) |  |
|  | Moderately and above | 12 (16.4) | 10 (18.5) |  |
| Lymphadenopathy |  |  |  | **0.007** |
|  | Not at All | 41 (56.2) | 44 (81.5) |  |
|  | Slightly | 22 (30.1) | 5 (9.3) |  |
|  | Moderately and above | 10 (13.7) | 5 (9.3) |  |
| Night Sweats |  |  |  | 0.081 |
|  | Not at All | 43 (58.9) | 42 (77.8) |  |
|  | Slightly | 21 (28.8) | 8 (14.8) |  |
|  | Moderately and above | 9 (12.3) | 4 (7.4) |  |
| Depression |  |  |  | 0.499 |
|  | Not at All | 50 (68.5) | 36 (66.7) |  |
|  | Slightly | 14 (19.2) | 14 (25.9) |  |
|  | Moderately and above | 9 (12.3) | 4 (7.4) |  |
| Fever |  |  |  | 0.797 |
|  | Not at All | 58 (79.5) | 43 (79.6) |  |
|  | Slightly | 9 (12.3) | 8 (14.8) |  |
|  | Moderately and above | 6 (8.2) | 3 (5.6) |  |
| Shortness of Breath |  |  |  | 0.680 |
|  | Not at All | 49 (67.1) | 34 (64.2) |  |
|  | Slightly | 19 (26.0) | 13 (24.5) |  |
|  | Moderately and above | 5 (6.8) | 6 (11.3) |  |
| Bone Pain |  |  |  | 0.405 |
|  | Not at All | 58 (79.5) | 37 (69.8) |  |
|  | Slightly | 10 (13.7) | 12 (22.6) |  |
|  | Moderately and above | 5 (6.8) | 4 (7.5) |  |
| Malaise |  |  |  | 0.294 |
|  | Not at All | 55 (75.3) | 38 (71.7) |  |
|  | Slightly | 14 (19.2) | 8 (15.1) |  |
|  | Moderately and above | 4 (5.5) | 7 (13.2) |  |
| Reduced Balance |  |  |  | 0.276 |
|  | Not at All | 48 (65.8) | 34 (63.0) |  |
|  | Slightly | 23 (31.5) | 15 (27.8) |  |
|  | Moderately and above | 2 (2.7) | 5 (9.3) |  |
| Weakness |  |  |  | **0.044** |
|  | Not at All | 50 (68.5) | 32 (59.3) |  |
|  | Slightly | 21 (28.8) | 14 (25.9) |  |
|  | Moderately and above | 2 (2.7) | 8 (14.8) |  |
| Bleeding/Bruising |  |  |  | 0.077 |
|  | Not at All | 54 (74.0) | 34 (63.0) |  |
|  | Slightly | 17 (23.3) | 13 (24.1) |  |
|  | Moderately and above | 2 (2.7) | 7 (13.0) |  |
| Weight Loss |  |  |  | 0.012 |
|  | Not at All | 61 (83.6) | 49 (89.1) |  |
|  | Slightly | 11 (15.1) | 1 (1.8) |  |
|  | Moderately and above | 1 (1.4) | 4 (7.4) |  |
| Nausea |  |  |  | 0.129 |
|  | Not at All | 71 (97.3) | 47 (88.7) |  |
|  | Slightly | 2 (2.7) | 5 (9.4) |  |
|  | Moderately and above | 0 (0.0) | 1 (1.9) |  |

**Supplementary Table 4.** Comparison of symptom scales for quality of life in CLL participants using EORTC QLQ-30 v3 and EORTC QLQ-CLL17 Questionnaire with treatment-naïve compared to treated CLL. All the scales range in scores from 0 to 100; a high score represents a higher (worse) level of symptoms.

|  | **Treatment Naïve** | **Treated** | **P value** |
| --- | --- | --- | --- |
|  | **Median (IQR)** | **Median (IQR)** |  |
| Fatigue | 22.2 (11.1-33.3) | 22.2 (0.0-44.4) | 0.308 |
| Nausea and Vomiting | 0.0 (0.0-0.0) | 0.0 (0.0-0.0) | 0.292 |
| Pain | 0.0 (0.0-15.7) | 0.0 (0.0-33.3) | **0.011** |
| Dyspnoea | 0.0 (0.0-33.3) | 0.0 (0.0-33.3) | 0.926 |
| Insomnia | 33.3 (0.0-33.3) | 33.3 (0.0-33.3) | 0.892 |
| Appetite Loss | 0.0 (0.0-0.0) | 0.0 (0.0-0.0) | 0.661 |
| Constipation | 0.0 (0.0-0.0) | 0.0 (0.0-0.0) | 0.441 |
| Diarrhoea | 0.0 (0.0-0.0) | 0.0 (0.0-0.0) | 0.354 |
| Financial Difficulties | 0.0 (0.0-0.0) | 0.0 (0.0-0.0) | 0.175 |
| Symptom Burden | 11.1 (0.0-19.4) | 11.11 (5.6-22.2) | 0.305 |
| Physical Condition | 15.6 (0.0-25.0) | 25.0 (8.3-41.7) | 0.075 |
| Worries/Fears | 71.4 (27.6-86.2) | 57.1 (16.7-76.2) | 0.093 |

**Supplementary Table 5.** Physical Activity (PA) Levels of CLL participants before and after diagnosis using Godin Leisure Time PA Questionnaire. PA is determined through the number of moderate and vigorous intensity units per week where ≥24 = active, 14-23 = Moderately Active, <14 = insufficiently active. Being active provides substantial health benefits, and being moderately active has some health benefits.

|  | **Activity Level Before Diagnosis (*N* = 117)** | **Current Physical Activity Levels**  **(*N* = 116)** | **P value** |
| --- | --- | --- | --- |
| Physical Activity Level, [N (%)] |  |  | 0.555 |
| Insufficient | 29 (24.8) | 32 (27.6) |  |
| Moderate | 14 (12.0) | 17 (14.7) |  |
| Active | 74 (63.2) | 67 (57.8) |  |

**Supplementary Table 6.** Before diagnosis and current physical activity levels of Treatment Naïve and Treated CLL participants using Godin Leisure Time Physical Activity Questionnaire.

|  | **Treatment Naïve** | | **Treated** | |  |
| --- | --- | --- | --- | --- | --- |
|  | **Before Diagnosis**  **(*N* = 68)** | **Current PA Levels**  **(*N* = 67)** | **Before Diagnosis**  **(*N* = 49)** | **Current PA Levels** | **P value** |
| Physical Activity Level, [N (%)] |  |  |  |  | 0.072 |
| Insufficient | 11 (16.2) | 11 (16.4) | 18 (36.7) | 21 (42.9) |  |
| Moderate | 9 (13.2) | 11 (16.4) | 5 (10.2) | 6 (12.2) |  |
| Active | 48 (70.6) | 45 (67.2) | 26 (53.1) | 22 (44.9) |  |

**Supplementary Table 7.** Comparison of global health status and functional scales of quality of life in CLL participants with different physical activity levels categorised based on the Godin Leisure Time Physical Activity Questionnaire. All the scales range in scores from 0 to 100; a high score represents a higher (better) level of functioning.

|  | **Insufficient** | **Moderate** | **Active** | **P value** |
| --- | --- | --- | --- | --- |
|  | **Median (IQR)** | **Median (IQR)** | **Median (IQR)** |  |
| Summary Score | 87.0 (76.4-94.8) | 85.83 (82.6-95.0) | 93.16 (84.3-97.0) | 0.126 |
| Global Health Status | 66.7 (47.9-83.3) | 83.3 (66.7-91.7) | 83.3 (66.7-91.7) | **0.024** |
| Physical Functioning | 86.7 (80.0-100.0) | 93.3 80.0-100.0) | 100.0 (93.3-100.0) | **0.003** |
| Role Functioning | 91.7 (66.7-100.0) | 100.0 (66.7-100.0) | 100.0 (100.0-100.0) | **0.020** |
| Emotional Functioning | 83.3 (64.6-100.0) | 100.0 (83.3-100.0) | 91.7 (75.0-100.0) | 0.079 |
| Cognitive Functioning | 83.3 (66.7-100.0) | 83.3 (75.0-100.0) | 100 (66.7-100.0) | 0.469 |
| Social Functioning | 83.3 (50.0-100.0) | 83.3 (58.4-100.0) | 100 (66.7-100.0) | 0.103 |

**Supplementary Table 8.** Comparison of symptom scales in quality of life in CLL participants with different physical activity Levels based on the Godin Leisure Time Physical Activity Questionnaire. All the scales range in scores from 0 to 100; a high score represents a higher (worse) level of symptoms.

|  | **Insufficient** | **Moderate** | **Active** | **P value** |
| --- | --- | --- | --- | --- |
|  | **Median (IQR)** | **Median (IQR)** | **Median (IQR)** |  |
| Fatigue | 27.7 (11.1-47.2) | 33.3 (22.2-38.9) | 11.1 (0.0-33.3) | **0.009** |
| Nausea and Vomiting | 0.0 (0.0-0.0) | 0.0 (0.0-0.0) | 0.0 (0.0-0.0) | 0.924 |
| Pain | 8.35 (0.0-33.3) | 0.0 (0.0-33.3) | 0.0 (0.0-0.0) | **0.012** |
| Dyspnoea | 0.0 (0.0-33.3) | 0.0 (0.0-33.3) | 0.0 (0.0-0.0) | 0.135 |
| Insomnia | 33.3 (0.0-66.7) | 33.3 (0.0-33.3) | 0.0 (0.0-33.3) | 0.141 |
| Appetite Loss | 0.0 (0.0-0.0) | 0.0 (0.0-0.0) | 0.0 (0.0-0.0) | 0.529 |
| Constipation | 0.0 (0.0-0.0) | 0.0 (0.0-0.0) | 0.0 (0.0-0.0) | 0.921 |
| Diarrhoea | 0.0 (0.0-0.0) | 0.0 (0.0-16.7) | 0.0 (0.0-0.0) | 0.708 |
| Financial Difficulties | 0.0 (0.0-33.3) | 0.0 (0.0-0.0) | 0.0 (0.0-0.0) | 0.275 |
| Symptom Burden | 16.7 (5.6-29.8) | 16.7 (5.6-22.2) | 5.6 (0.0-16.7) | **0.019** |
| Physical Condition | 25.0 (8.3-43.8) | 25.0 (4.2-25.0) | 16.7 (0.0-25.0) | **0.028** |
| Worries/Fears | 71.4 (19.0-81.0) | 38.1 (16.7-64.3) | 61.9 (23.8-81.0) | 0.284 |

Supplementary Figure 1. PatientQ-CLL questionnaire

We collected data from this cross-sectional observation study using an online version of a 183-part questionnaire separated into nine sections (Supplementary Figure 1). Briefly, the questionnaire captured:

Section 1 (Items 1-8) – Personal Information. Participant demographics, current living situation, mode of transport to medical consultations, and highest level of education.

Section 2 (Items 9-33) – Self-reported health- and disease-related characteristics. Date of diagnosis, current treatment stage; Treatment-Naïve, individuals who have not received treatment and are currently going through a period of observation (Active Monitoring); In Treatment, individuals who are currently undergoing active treatment for CLL; Post-Treatment, individuals who have previously undergone treatment for CLL but are currently in remission [2], current and previous treatments, current healthcare; private or National Health Service (NHS), and severity of CLL-related symptoms experienced in the past month (classified as ‘not at all’, ‘slightly’, ‘moderately’, ‘severely’ and ‘overwhelmingly’).

Section 3 (Items 34-41) – Self-reported physical activity levels. Participants were presented with two separate validated and reliable physical activity questionnaires. The Stanford Brief Activity Survey (SBAS) [19, 20] and the Godin Leisure Time Physical Activity Questionnaire (Godin LTPAQ) [21]. The Godin LTPAQ is commonly used to classify individuals into active and insufficiently active categories, with a moderate correlation between Godin LTPAQ and a pedometer step count in leukaemia and other cancer survivors having been observed [22, 23]. The Godin LTPAQ was used to understand current physical activity levels; however, the questionnaire was also adapted to gauge an understanding of physical activity levels before diagnosis. Godin LTPAQ physical activity categories were determined by moderate and vigorous intensity units per week where ≥24 units were classified as “Active”, 14-23 units as “Moderately Active”, and <14 units as “Insufficiently Active”. However, when categorising into whether participants met physical activity guidelines or not, the time spent in moderate and vigorous activity was summed, with the minutes in vigorous activity weighted by two to account for greater intensity. Those who scored 150 minutes per week or greater were categorised as meeting physical activity guidelines [24, 25].

Section 4 (Items 42-75) – Interest and advice on physical activity. Participants were questioned on the physical activity advice and guidance they had received from their healthcare professionals, their interest in participating in an exercise programme and their preferences regarding the delivery of an exercise programme. Responses were collected using a 5-point Likert scale ranging from 1 (strongly disagree/very unimportant) to 5 (strongly agree/very important). The questions used in this section were based on previous research into how individuals with Multiple Myeloma, a blood cancer with similar symptoms to CLL (e.g. fatigue, muscle weakness), view exercise and their preferences for participating in physical activities [24].

Section 5 (Items 76-121) – Self-reported quality of life (QoL). Participants were asked questions from the English version of the European Organisation for Research and Treatment of Cancer Quality of Life Questionnaires - Core30 (EORTC QLQ-C30 v3) [26, 27], including the extension for CLL (EORTC QLQ-CLL17) [28]. All scales range in scores from 0 to 100; a high score represents a higher (better) level of functioning or a higher (worse) level of symptoms. [29].

Section 6 (Items 122-151) – CLL Comorbidities Index. Participants were presented with questions on their present and past medical status to understand their comorbidity burden. Questions covered cardiac, vascular, haematological, respiratory, EENT (eyes, ear, nose, throat, larynx), upper/lower GI, hepatic, pancreatic, renal, genitourinary, musculoskeletal, neurological, endocrine-metabolic, and psychiatric/Behavioural conditions. Each condition was then scored using the Cumulative Illness Rating Scales [30] adapted to CLL [31]. The sum of the system scores creates an overall comorbidity index.

Section 7 (Items 152-176) - Dietary Analysis. To evaluate diet quality, participants responded to questions from a short-form food frequency questionnaire (SFFFQ) [32]. A dietary quality score (DQS) was derived from their intake of fruits, vegetables, oily fish, non-milk extrinsic sugars, and fats, which are considered key indicators of a healthy diet. For each component, scores ranged from 1 to 3, with a score of 3 indicating adherence to UK dietary guidelines for that food group. The DQS ranged from a minimum of 5 to a maximum of 15, representing the optimal intake of these foods. This questionnaire has been validated by comparing it with a comprehensive food frequency questionnaire (FFQ), showing a significant agreement in DQS between the SFFFQ and FFQ within a UK population [32].

Section 8 (Items 177-183) - Additional Self-reported Health and Disease Characteristics. This section included information that not all participants may have had access to or know, which could have posed a barrier to completing the questionnaire. Questions included recent medical test results (i.e., Complete blood count results and Cytogenic Results [2]) and anaemia diagnosis. It was clear to the participants that these questions were optional.

Start of Block: Participant Information Sheet

  Thank you for considering taking part in this research.    Please read the participant information sheet below before proceeding with the questionnaire. Please also download a copy of the information sheet for your records using the link below: PatientQ-CLL Information Sheet

Start of Block: Section 1: Personal Information

**Personal Information**
Within the section,  you will be asked for information regarding your demographics.

1
What is your sex?

- Male (1)
- Female (2)
- Prefer not to say (3)

2 What is your weight? Please answer in your preferred metric: kilograms or stones and pounds

- in kilograms (kg) (4) __________________________________________________
- in stones and pounds (5) __________________________________________________

3 How tall are you? Please answer in your preferred metric: centimetres or feet and inches

- in centimetres (cm) (1) __________________________________________________
- in feet and inches (2) __________________________________________________

4 What is your Ethnicity?

▼ Arab (1) ... Other (Please State) (23)

5 Who do you live with? (please select all that are relevant)

- with Partner/Spouse (1)
- with Child/Children (under 18) (2)
- with a finically dependent adult (e.g. student) (4)
- with an independent adult (7)
- with extended family/friends (8)
- Alone (9)

6 What is your area of residence?

- Urban (e.g. Regions surrounding a city) (1)
- Rural (e.g. countryside located outside towns and cities) (2)

7 What mode of transport do you use to get to medical appointments (Please select all that are relevant)?

- Personally own vehicle (e.g. car/van/motorbike) (1)
- Public transport (e.g. bus/train) (2)
- Private Vehicle (e.g. Taxi) (3)
- Bicycle (4)
- By Foot (5)
- Other (please state) (6) __________________________________________________

8 What is your highest level of education?

▼ Secondary School to Year 11 (1) ... University Postgraduate Research Degree (e.g. PhD) (6)

End of Block: Section 1: Personal Information

Start of Block: Section 2 : Self-reported health and disease related characteristics

**Self-reported health- and disease-related characteristics**

During this section, you will be asked questions regarding your health which we understand can be a sensitive topic for some. If you feel distressed at any point, please contact a research team member so we can provide you with the necessary support.  

Additionally, if you do not know this information, please do not worry and select 'I do not know'.
 

1 What date were you diagnosed with CLL/SLL? (If you don't know the exact date please put, 01/mm/yyyy)

- Date (DD/MM/YYYY) (1) __________________________________________________

2 What is your current clinical stage?

- Binet Stage A (1)
- Binet Stage B (2)
- Binet Stage C (3)
- I do not know (8)

8 What treatment stage are you in?

- Treatment Naive (Watch and Wait or Active Monitoring) (1)
- In Treatment (2)
- Post Treatment (3)

Skip To: 12 If What treatment stage are you in? = Treatment Naive (Watch and Wait or Active Monitoring)

Skip To: 10 If What treatment stage are you in? = Post Treatment

9 What CLL/SLL treatment are you currently on? (Please select all that are relevant)

- FCR (Fludarabine/Cyclophosphamide/Rituximab) Chemo-immunotherapy (1)
- Rituximab (6)
- Ibrutinib (7)
- Venetoclax (Venclyxto) (9)
- Obinutuzumab (11)
- Ofatumumab (8)
- Acalabrutinib (10)
- Other (please state) (12) __________________________________________________
- I do not know (13)

10 What CLL/SLL treatment have you received? (Please select all that are relevant)

- FCR (Fludarabine/Cyclophosphamide/Rituximab) Chemo-immunotherapy (1)
- Rituximab (6)
- Ibrutinib (7)
- Venetoclax (Venclyxto) (9)
- Obinutuzumab (8)
- Ofatumumab (5)
- Acalabrutinib (10)
- Other (please state) (12) __________________________________________________
- No other medication (13)
- I do not know (14)

11 How many years (roughly) have you been/were you on treatment?

________________________________________________________________

12 What healthcare do you have currently?

- Private (1)
- NHS (4)

| Page Break |  |
| --- | --- |

13 Please rate the intensity of each symptom related to CLL/SLL you have experienced in the last month (classified as not at all, slightly, moderately, severe and overwhelming)

|  | Not at all (1) | Slightly (2) | Moderately (3) | Severe (4) | Overwhelming (10) |
| --- | --- | --- | --- | --- | --- |
| Tiredness/Fatigue (1) |  |  |  |  |  |
| Enlarged lymph nodes (lymphadenopathy) (15) |  |  |  |  |  |
| Infections (urinary tract infection, upper respiratory tract infection, skin infection and chest infection symptoms) (16) |  |  |  |  |  |
| Night sweats (17) |  |  |  |  |  |
| Excessive bleeding/bruising (18) |  |  |  |  |  |
| Weakness (19) |  |  |  |  |  |
| Shortness of breath (20) |  |  |  |  |  |
| Unintentional weight loss (21) |  |  |  |  |  |
| Fever (22) |  |  |  |  |  |
| Depression (23) |  |  |  |  |  |
| Anxiety (24) |  |  |  |  |  |
| Stress (25) |  |  |  |  |  |
| Lack of sleep/insomnia (26) |  |  |  |  |  |
| Reduced balance (27) |  |  |  |  |  |
| Bone pain (28) |  |  |  |  |  |
| Vomiting and Nausea (31) |  |  |  |  |  |
| Malaise (general feeling of being ill) (32) |  |  |  |  |  |
| Another relevant symptom (please state) (33) |  |  |  |  |  |

End of Block: Section 2: Self-reported health and disease related characteristics

Start of Block: Section 3: Physical Activity Levels

**Physical Activity Levels**
 We are now going to present to you two different physical activity questionnaires, please answer all questions in all questionnaires.

**Physical Activity Questionnaire 1**

1 **During a typical 7-day period (a week)** before diagnosis**, how many times did/do you do the following kind of exercise for more than 15 minutes during your free time. (Please only type a number in the boxes below)**

  Strenuous Exercise (heart beats rapidly) (e.g., running, jogging, hockey, rugby, football, squash, basketball, cross country skiing, judo, roller skating, vigorous swimming, vigorous long-distance bicycling)

________________________________________________________________

  Moderate Exercise (not exhausting) (e.g., fast walking, baseball, tennis, easy bicycling, volleyball, badminton, easy swimming, alpine skiing, popular and folk dancing, brisk walk)

________________________________________________________________

  Mild/light exercise (minimal effort) (e.g., yoga, archery, fishing, bowling, golf, easy walking, plates)

________________________________________________________________

2 **During a typical 7-day period (a week) after your diagnosis (before treatment), how many times did/do you do the following kind of exercise for more than 15 minutes during your free time. (Please only type a number in the boxes below)**

  Strenuous Exercise (heart beats rapidly) (e.g., running, jogging, hockey, rugby, football, squash, basketball, cross country skiing, judo, roller skating, vigorous swimming, vigorous long-distance bicycling)

________________________________________________________________

  Moderate Exercise (not exhausting) (e.g., fast walking, baseball, tennis, easy bicycling, volleyball, badminton, easy swimming, alpine skiing, popular and folk dancing, brisk walk)

________________________________________________________________

  Mild/light exercise (minimal effort) (e.g., yoga, archery, fishing, bowling, golf, easy walking, plates)

________________________________________________________________

**Physical Activity Questionnaire 2**

1 **On-The-Job Activity** Please check the box next to the one statement that best describes the kinds of physical activity you usually performed while on the job this last year. If you are not gainfully employed outside the home but perform work around the home regularly, indicate that activity in this section.

- A. If you have no job or regular work, check Box A and go on to Question 2. (1)
- B. I spent most of the day sitting or standing. When I was at work I did such things as writing, typing, talking on the telephone, assembling small parts or operating a machine that takes very little exertion or strength. If I drove a car or truck while at work, I did not lift or carry anything for more than a few minutes each day. (2)
- C. I spent most of the day walking or using my hands and arms in work that required moderate exertion. When I was at work, I did such things as delivering mail, patrolling on guard duty, mechanical work on automobiles or other large machines, house painting or operating a machine that requires some moderate activity. If I drove a truck or lift, my job required me to lift and carry things frequently. (3)
- D. I spent most of the day lifting or carrying heavy objects or moving most of my body in some other way. When I was at work, I did such things as stacking cargo or inventory, handling parts or materials, or I did work like that of a carpenter who builds structures or a gardener who does most of the work without machines. (4)
- E. I spent most of the day doing hard physical labor. When I was at work, I did such things as digging or chopping with heavy tools or carrying heavy loads (bricks, for example) to the place where they are to be used. If I drove a truck or operating equipment, my job also required me to do hard physical work most of the day with only short breaks (5)

2 **Leisure-Time Activity** Please check the box next to the one statement which best describes the way you spent your leisure time during most of the last year.

- A. Most of my leisure time was spent without very much physical activity. I mostly did things like watching television, reading or playing cards. If I did anything else, it was likely to be light chores around the house or yard or some easy-going game like bowling or catch. Only occasionally, no more than once or twice a month, did I do anything more vigorous, like jogging, playing tennis or active gardening. (1)
- B. On Weekdays, when I got home from work, I did a few active things. But most weekends I was able to get outdoors for some light exercise- going for walks, playing a round of golf (without motorised carts), or doing some active chores around the house (2)
- C. Three times per week, on average, I engaged in some moderate activity- such as brisk walking or slow jogging, swimming or riding a bike for 15-20 minutes or more. Or I spent 45 minutes to an hour or more doing moderately difficult chores such as raking or washing windows, mowing the lawn or vacuuming or playing games such as doubles tennis or basketball. (3)
- D. During my leisure time over the past year, I engaged in a regular program of physical fitness involving some kind of heavy physical activity at least three times per week. Examples of heavy physical activity are jogging, running or riding fast on a bicycle for 30 minutes or more; heavy gardening or other chores for an hour or more; active games or sports such as handball or tennis for an hour or more; or a regular program involving calisthenics and jogging or the equivalent for 30 minutes or more. (4)
- E. Over the past year I engaged in a regular program of physical fitness along the lines described in the last paragraph (I), but I did it almost daily- five or more times per week. (5)

End of Block: Section 3: Physical Activity Levels

Start of Block: Section 4: Interest and advice on physical activity

**Interest and advice on physical activity** Within this section, we will ask you questions regarding what physical activity advice you have received and from whom as well as your preferences on physical activity programming.

1 Have you received PA and exercise advice since your diagnosis of CLL/SLL from a health care professional e.g. CLL/SLL specialist, rehabilitation specialist, general practitioner, physiotherapist, exercise physiology, nurse, or consultant?

- Yes (1)
- No (2)
- Unsure (3)

2 Have you ever been recommended by your CLL specialist to see an exercise specialist e.g. exercise physiologist or physiotherapist for PA and exercise advice?

- Never (1)
- Rarely (2)
- Occasionally (3)
- Always (4)
- Only when asked (5)

3 Would you be interested in attending an exercise programme for people with CLL/SLL?

- Yes (1)
- No (2)

  If no, why not?

________________________________________________________________

________________________________________________________________

________________________________________________________________

________________________________________________________________

________________________________________________________________

4 Please indicate your agreement levels to the statements below: Exercise programmes should be prescribed during...

|  | Strongly Disagree (1) | Disagree (2) | Neutral (3) | Agree (7) | Strongly Agree (8) |
| --- | --- | --- | --- | --- | --- |
| treatment naive, active treatment and post treatment (1) |  |  |  |  |  |
| treatment-naive and active treatment only (6) |  |  |  |  |  |
| treatment-naive and post-treatment only (7) |  |  |  |  |  |
| active treatment and post treatment only (8) |  |  |  |  |  |
| treatment naive only (9) |  |  |  |  |  |
| active treatment only (10) |  |  |  |  |  |
| post treatment only (28) |  |  |  |  |  |

  Exercise programmes should be led by....

|  | Strongly Disagree (1) | Disagree (2) | Neutral (3) | Agree (4) | Strongly Agree (5) |
| --- | --- | --- | --- | --- | --- |
| physios with an interest in cancer (1) |  |  |  |  |  |
| exercise physiologist with an interest in cancer (4) |  |  |  |  |  |
| a personal trainer (5) |  |  |  |  |  |
| a CLL/SLL patient (6) |  |  |  |  |  |

  Exercise programmes should be delivered...

|  | Strongly Disagree (1) | Disagree (2) | Neutral (3) | Agree (4) | Strongly Agree (5) |
| --- | --- | --- | --- | --- | --- |
| in small groups with any cancer (7) |  |  |  |  |  |
| in Small groups with CLL/SLL and carers (8) |  |  |  |  |  |
| as 1-2-1 sessions only (9) |  |  |  |  |  |
| as 1-2-1 with CLL and partner only (10) |  |  |  |  |  |
| in groups with the general public (11) |  |  |  |  |  |

  Exercise programmes should be delivered and completed

|  | Strongly Disagree (1) | Disagree (2) | Neutral (3) | Agree (4) | Strongly Agree (5) |
| --- | --- | --- | --- | --- | --- |
| by DVD in own home (7) |  |  |  |  |  |
| by live virtual classes via online platform (12) |  |  |  |  |  |
| at the hospital where treatment is prescribed (13) |  |  |  |  |  |
| at a community clinic/gym (14) |  |  |  |  |  |
| at a private practice for physio/exercise physiologist (15) |  |  |  |  |  |
| as a programme to do in your own time with limited guidance (16) |  |  |  |  |  |

5 Please indicate the importance of the below statements

|  | Very Unimportant (1) | Unimportant (2) | Neutral (3) | Important (4) | Very Important (5) |
| --- | --- | --- | --- | --- | --- |
| Having an exercise program with flexible times (7) |  |  |  |  |  |
| Having an exercise programme with low cost (12) |  |  |  |  |  |
| Facilities where parking is always available (13) |  |  |  |  |  |
| Location of facilities close to home (less than 30 minute drive away) (14) |  |  |  |  |  |
| Facilities with good transport links (15) |  |  |  |  |  |
| Facilities with good COVID-19 Protocols (16) |  |  |  |  |  |

Are you involved or have been involved in any Clinical Trial involving exercise? If so, please state the Clinical Trial l and dates you started and finished the trial below. If you haven't been involved in a clinical trial, please leave the below blank.

________________________________________________________________

________________________________________________________________

________________________________________________________________

________________________________________________________________

________________________________________________________________

If you would like to express any additional thoughts, please use the text entry box below:

________________________________________________________________

________________________________________________________________

________________________________________________________________

________________________________________________________________

________________________________________________________________

End of Block: Section 4 : Interest and advice on physical activity

Start of Block: Section 5 : Quality of Life Questionnaire

**Quality of Life Questionnaire** During this section, you will be asked questions regarding your health, daily activity and general well-being within the last week and the last month.

1 For each statement below, please select the point that best applies to you. There are no ‘right’ or 'wrong' answers.

|  | Not at all (1) | A Little (2) | Quite a bit (3) | Very much (4) |
| --- | --- | --- | --- | --- |
| Do you have any trouble doing strenuous activities, like carrying a heavy shopping bag or a suitcase? (1) |  |  |  |  |
| Do you have any trouble taking a long walk? (5) |  |  |  |  |
| Do you have any trouble taking a short walk outside of the house? (6) |  |  |  |  |
| Do you need to stay in bed or a chair during the day? (7) |  |  |  |  |
| Do you need help with eating, dressing, washing yourself or using the toilet? (8) |  |  |  |  |

  During the past week:

|  | Not at all (1) | A Little (2) | Quite a bit (3) | Very much (4) |
| --- | --- | --- | --- | --- |
| Were you limited in doing either your work or other daily activities? (1) |  |  |  |  |
| Were you limited in pursuing your hobbies or other leisure time activities? (4) |  |  |  |  |
| Were you short of breath? (5) |  |  |  |  |
| Have you had pain? (6) |  |  |  |  |
| Did you need to rest? (7) |  |  |  |  |
| Have you had trouble sleeping? (8) |  |  |  |  |
| Have you felt weak? (9) |  |  |  |  |
| Have you lacked appetite? (10) |  |  |  |  |
| Have you felt nauseated? (11) |  |  |  |  |
| Have you vomited? (12) |  |  |  |  |
| Have you been constipated? (13) |  |  |  |  |
| Have you had diarrhoea? (14) |  |  |  |  |
| Were you tired? (15) |  |  |  |  |
| Did pain interfere with your daily activities? (16) |  |  |  |  |
| Have you had difficulty in concentrating on things, like reading a newspaper or watching television? (17) |  |  |  |  |
| Did you feel tense? (18) |  |  |  |  |
| Did you worry? (19) |  |  |  |  |
| Did you feel irritable? (20) |  |  |  |  |
| Did you feel depressed? (21) |  |  |  |  |
| Have you had difficulty remembering things? (22) |  |  |  |  |
| Has your physical condition or medical treatment interfered with your family life? (23) |  |  |  |  |
| Has your physical condition or medical treatment interfered with your social activities? (24) |  |  |  |  |
| Has your physical condition or medical treatment caused you financial difficulties? (25) |  |  |  |  |

2 For the following question please select the number between 1 and 7 that best applies to you, where 1 is very poor and 7 is excellent.

|  | Very Poor 1 (1) | 2 (2) | 3 (3) | 4 (4) | 5 (5) | 6 (6) | Excellent 7 (7) |
| --- | --- | --- | --- | --- | --- | --- | --- |
| How would you rate your overall health during the past week? (1) |  |  |  |  |  |  |  |
| How would you rate your overall quality of life during the past week? (8) |  |  |  |  |  |  |  |

3 For the following questions please indicate the extent to which you have experienced these symptoms or problems. Please select the number that best applies to you. During the past week:

|  | Not at all (1) | A Little (2) | Quite a bit (3) | Very Much (4) |
| --- | --- | --- | --- | --- |
| Have you had muscle weakness? (1) |  |  |  |  |
| Have you had aches or pains in your muscles or joints? (5) |  |  |  |  |
| Have you had aches or pains in your bones? (6) |  |  |  |  |
| Have you had a dry mouth? (7) |  |  |  |  |
| Have you felt ill or unwell? (8) |  |  |  |  |
| Have you had night sweats? (9) |  |  |  |  |
| Have you had shortness of breath on exertion? (10) |  |  |  |  |
| Have you had a lack of energy? (11) |  |  |  |  |
| Have you felt drowsy? (12) |  |  |  |  |
| Have you had sudden tiredness? (13) |  |  |  |  |

During the past 4 weeks:

|  | No at all (1) | A Little (2) | Quite a bit (3) | Very Much (4) |
| --- | --- | --- | --- | --- |
| Have you worried about your health in the future? (1) |  |  |  |  |
| Have you worried about the recurrence of your disease? (4) |  |  |  |  |
| Have you worried about becoming dependent on others? (5) |  |  |  |  |
| Have you worried about getting another type of cancer? (6) |  |  |  |  |
| Have you worried about your treatment causing future health problems? (7) |  |  |  |  |
| If applicable: Have you had problems at your work or place of study due to the disease? (8) |  |  |  |  |
| If applicable: Have you worried about not being able to continue working or your education? (9) |  |  |  |  |

End of Block: Section 5 - Quality of Life Questionnaire

Start of Block: Section 6 - Comorbidities Index

**Co-Morbidities Index** During this section, you will be asked about any other health conditions you may have; please answer honestly and to the best of your ability. This questionnaire is to help us distinguish between CLL and other co-morbidities and their effect on your quality of life and physical activity levels.  We understand this can be a sensitive topic for some. If you feel distressed at any point, please contact a research team member so we can provide you with the necessary support. If you are unsure of any information, please just give your best description and move on to the next question.

1 Do you currently have or every had any cardiac conditions (e.g. angina, heart attack, abnormal heart rhythm, valve problems)?

- Yes (Please state condition and diagnosis year and years with condition e.g. Angina - diagnosed 03/2022) (1) __________________________________________________
- No (2)

Display This Question:

If Do you currently have or every had any cardiac conditions (e.g. angina, heart attack, abnormal he... != No

  Are you being treated or have you been treated for your condition/s?

- Yes (please describe treatment e.g. medication taken daily/occasionally/when symptomatic, have you had surgery etc) (1) __________________________________________________
- No (2)

2 Do you currently have or had any circulatory/vascular conditions independent of CLL/SLL (e.g. peripheral atherosclerotic disease, aneurysm of the abdominal aorta, deep vein thrombosis, hypertension, high cholesterol)?

- Yes (Please state condition and diagnosis year and years with condition e.g. Deep vein thrombosis - diagnosed 03/2022) (1) __________________________________________________
- No (2)

Display This Question:

If Do you currently have or had any circulatory/vascular conditions independent of CLL/SLL (e.g. per... != No

  Are you being treated or have been treated for your condition/s?

- Yes (please describe treatment e.g. medication taken daily/occasionally/when symptomatic, have you had surgery etc) (1) __________________________________________________
- No (2)

3 Do you currently have any blood conditions that are not related to your CLL/SLL (e.g. Sepsis or sickle cell anaemia)?

- Yes (Please state condition and diagnosis year and years with condition e.g. Sepsis - diagnosed 03/2022) (1) __________________________________________________
- No (2)

Display This Question:

If Do you currently have any blood conditions that are not related to your CLL/SLL (e.g. Sepsis or s... != No

  Are you being treated or have you been treated for your condition/s?

- Yes (please describe treatment e.g. medication taken daily/occasionally/when symptomatic, have you had surgery etc) (1) __________________________________________________
- No (2)

4 Do you currently have any respiratory conditions (e.g. asthma, emphysema, COPD, lung cancer)?

- Yes (Please state condition and diagnosis year and years with condition e.g. Asthma - diagnosed 03/2022) (1) __________________________________________________
- No (2)

Display This Question:

If Do you currently have any respiratory conditions (e.g. asthma, emphysema, COPD, lung cancer)?  != No

  Are you being treated or have you been treated for your condition/s?

- Yes (please describe treatment e.g. medication taken daily/occasionally/when symptomatic, have you had surgery etc) (1) __________________________________________________
- No (2)

  Do you currently smoke?

- Yes (please state the average amount (in packs) of cigarettes you have per day and how many years have you smoked for) e.g 1 pack a day for 20 years (1) __________________________________________________
- No (2)

5 Do you currently have or have had any conditions of the eyes (e.g. glaucoma, cataract, important loss of vision), ears (including important hearing impairment), nose, throat or voice?  Note: vertigo and dizziness are included in this section unless they are of neurological origin

- Yes (Please state condition and diagnosis year and years with condition e.g. cataract - diagnosed 03/2022) (1) __________________________________________________
- No (2)

Display This Question:

If Do you currently have or have had any conditions of the eyes (e.g. glaucoma, cataract, important... != No

  Are you being treated or have you been treated for your condition/s?

- Yes (please describe treatment e.g. medication taken daily/occasionally/when symptomatic, have you had surgery etc) (1) __________________________________________________
- No (2)

6 Do you currently have or have had any upper stomach or digestion conditions? (e.g. Stomach Ulcers, Acid Reflux)

- Yes (Please state condition and diagnosis year and years with condition e.g. Stomach Ulcers - diagnosed 03/2022) (1) __________________________________________________
- No (2)

Display This Question:

If Do you currently have or have had any upper stomach or digestion conditions? (e.g. Stomach Ulcers... != No

  Are you being treated or have been treated for your condition/s?

- Yes (please describe treatment e.g. medication taken daily/occasionally/when symptomatic, have you had surgery etc) (1) __________________________________________________
- No (2)

7 Do you currently have or have had any intestinal or bowel conditions (e.g. intestinal hernias, constipation, colorectal cancer and incontinence)?

- Yes (Please state condition and diagnosis year and years with condition e.g. incontinence- diagnosed 03/2022) (1) __________________________________________________
- No (2)

Display This Question:

If Do you currently have or have had any intestinal or bowel conditions (e.g. intestinal hernias, co... != No

  Are you being treated or have been treated for your condition/s?

- Yes (please describe treatment e.g. medication taken daily/occasionally/when symptomatic, have you had surgery etc) (1) __________________________________________________
- No (2)

8 Do you currently have or have had any liver or pancreas conditions?

- Yes (Please state condition and diagnosis year and years with condition e.g. Liver disease - diagnosed 03/2022) (1) __________________________________________________
- No (2)

Display This Question:

If Do you currently have or have had any liver or pancreas conditions? != No

  Are you being treated or have you been treated for your condition/s?

- Yes (please describe treatment e.g. medication taken daily/occasionally/when symptomatic, have you had surgery etc) (1) __________________________________________________
- No (2)

9 Do you currently have or have you had any kidney conditions (e.g. chronic kidney disease, reoccurring infection)?

- Yes (Please state condition and diagnosis year and years with condition e.g. kidney disease - diagnosed 03/2022) (1) __________________________________________________
- No (2)

Display This Question:

If Do you currently have or have you had any kidney conditions (e.g. chronic kidney disease, reoccur... != No

  Are you being treated or have you been treated for your current condition/s?

- Yes (please describe treatment e.g. medication taken daily/occasionally/when symptomatic, have you had surgery etc) (1) __________________________________________________
- No (2)

10 Do you currently have any urinary conditions (e.g. incontinence, reoccurring infection)?

- Yes (Please state condition and diagnosis year and years with conditong e.g. incontinence - diagnosed 03/2022) (1) __________________________________________________
- No (2)

Display This Question:

If Do you currently have any urinary conditions (e.g. incontinence, reoccurring infection)? != No

  Are you being treated for your current condition/s?

- Yes (please describe treatment e.g. medication name, taken daily/occasionally/when symptomatic, date you started, surgery etc) (1) __________________________________________________
- No (2)

11 Do you currently have or had any condition of the skin, joints, bones or muscles (e.g. osteoporosis, arthritis, and any other skin or musculoskeletal conditions)?

- Yes (Please state condition and diagnosis year and years with condition e.g. osteoporosis- diagnosed 03/2022) (1) __________________________________________________
- No (2)

Display This Question:

If Do you currently have or had any condition of the skin, joints, bones or muscles (e.g. osteoporos... != No

  Are you being treated or have you been treated for your current condition/s e.g. anti-inflammatory drugs for these problems? Creams prescribed by doctors?

- Yes (please describe treatment e.g. medication taken daily/occasionally/when symptomatic, have you had surgery etc) (1) __________________________________________________
- No (2)

2 Do you currently have any neurological conditions (e.g. peripheral neuropathy, headaches, nerve damage)?

- Yes (Please state condition and diagnosis years and years with condition e.g. peripheral neuropathy - diagnosed 03/2022) (1) __________________________________________________
- No (2)

Display This Question:

If Do you currently have any neurological conditions (e.g. peripheral neuropathy, headaches, nerve d... != No

  Are you being treated or have you been treated for your condition/s?

- Yes (please describe treatment e.g. medication taken daily/occasionally/when symptomatic, date you started, surgery etc) (1) __________________________________________________
- No (2)

13 Do you currently have any metabolic conditions (e.g, thyroid gland conditions, diabetes, breast or prostate cancer, or any other hormonal problems)?

- Yes (Please state condition and diagnosis years and years with condition e.g. diabetes - diagnosed 03/2022) (1) __________________________________________________
- No (2)

Display This Question:

If Do you currently have any metabolic conditions (e.g, thyroid gland conditions, diabetes, breast o... != No

  Are you being treated or have you been treated for your condition/s?

- Yes (please describe treatment e.g. medication taken daily/occasionally/when symptomatic, have you had surgery etc) (1) __________________________________________________
- No (2)

   Are you currently experiencing/have experienced menopause or andropause?

- Yes - non-symptomatic and without hormonotheraphy (1)
- Yes - symptomatic or with hormonotherapy (2)
- No (3)

14 Do you currently have depression, anxiety or other psychiatric/behavioral conditions?

- Yes (Please state condition and diagnosis years and years with condition e.g. depression - diagnosis 03/2022) (1) __________________________________________________
- No (2)

Display This Question:

If Do you currently have depression, anxiety or other psychiatric/behavioral conditions? != No

  Are you being treated or have you been treated for your current condition/s?

- Yes (please describe treatment e.g. medication taken daily/occasionally/when symptomatic, have you had surgery etc) (1) __________________________________________________
- No (2)

End of Block: Section 6 - Comorbidities Index

Start of Block: Section 7 - Dietary Analysis

**The next two sections (Dietary Analysis and Additional Self-reported health and disease characteristics) are OPTIONAL.** **If you don't want to complete this section please scroll down to the bottom of the page and click the >> button until you have a 'thank you for completing the questionnaire' message appear**

**Dietary Analysis - Optional**  During this section, you will be asked questions regarding your diet. Please answer to your best ability.   **If you don't want to complete this section, please scroll down to the bottom of the page and click the >> button until the next section (Additional Self-reported health and disease characteristics) appears.**   Pick and select how often you eat at least ONE portion of the following foods and drinks: (a portion includes a handful of grapes, an orange, a serving of carrots, a side salad, a slice of bread, and a glass of pop).   (please only select one, but answer EVERY line)

|  | Rarely or Never (1) | Less than 1 a week (2) | Once a week (3) | 2-3 times a week (4) | 4-6 times a week (5) | 1-2 times a day (6) | 3-4 times a day (7) | 5+ a day (8) |
| --- | --- | --- | --- | --- | --- | --- | --- | --- |
| Fruit (tinned/fresh) (1) |  |  |  |  |  |  |  |  |
| Fruit Juice (not cordial or squash) (2) |  |  |  |  |  |  |  |  |
| Salad (not garnish added to sandwiches) (3) |  |  |  |  |  |  |  |  |
| Vegetables (tinned/frozen/fresh but not potatoes) (4) |  |  |  |  |  |  |  |  |
| Chips/ fried potatoes (5) |  |  |  |  |  |  |  |  |
| Beans or pulses like baked beans, chickpeas, dahl (6) |  |  |  |  |  |  |  |  |
| Fibre-rich breakfast cereal, like Weetabix, Fruit 'n Fibre, Porridge, Muesli (7) |  |  |  |  |  |  |  |  |
| Wholemeal bread or chapattis (8) |  |  |  |  |  |  |  |  |
| Cheese / Yoghurt (9) |  |  |  |  |  |  |  |  |
| Crisps / Savoury Snacks (10) |  |  |  |  |  |  |  |  |
| Sweet biscuits, cakes, chocolate, sweets (11) |  |  |  |  |  |  |  |  |
| Ice Cream / cream (12) |  |  |  |  |  |  |  |  |
| Non alcoholic fizzy drinks/pop (not sugar free or diet) (13) |  |  |  |  |  |  |  |  |
| Beef, Lamb, Pork, Ham - steaks, roasts, joints, mince or chops (14) |  |  |  |  |  |  |  |  |
| Chicken or Turkey - steaks, roasts, joints, mince or portions (not in batter or breadcrumbs) (15) |  |  |  |  |  |  |  |  |
| Sausages, bacon, corned beef, meat pies/pasties, burgers (16) |  |  |  |  |  |  |  |  |
| Chicken/turkey nuggets/twizzlers, turkey burgers, chicken pies or in batter or breadcrumbs (17) |  |  |  |  |  |  |  |  |
| White fish in batter or breadcurms - like 'fish ', chips' (18) |  |  |  |  |  |  |  |  |
| White fish not in batter or breadcrumbs (19) |  |  |  |  |  |  |  |  |
| Oily fish - like herrings, sardines, salmon, trout, mackerel, fresh tuna (not tinned tuna) (20) |  |  |  |  |  |  |  |  |

On average, how many portions of FRUIT do you eat a day? (examples include a handful of graphs, an orange, a glass of fruit juice, a handful of dried fruits)

________________________________________________________________

On average, how many portions of VEGETABLES do you eat a day? (examples include: 3 heaped tablespoons of carrots, a side salad, 2 spears of broccoli)

________________________________________________________________

What supplements do you take, if any? (Please select all that are relevant)

- Iron (1)
- B12 (2)
- Folate (3)
- Vitamin D (6)
- Other (please state) (4) __________________________________________________
- None (5)

What milk do you usually use or drink, such as in hot & cold drinks or on cereal? (including tea, coffee, hot milk, milk shakes, or on cereal)

- Whole/full-fat milk (1)
- Semi-skimmed milk (2)
- Skimmed milk (3)
- Rarely/never use milk (4)
- Other (please state) (5) __________________________________________________

On average, how much alcohol do you drink over a complete seven-day week? (one unit is a standard glass of wine, half a pint of beer or lager, a single measure of spirits, a measure of sherry)

- I rarely/never drink alcohol (1)
- Less than 14 units (2)
- between 14 & 21 units (3)
- More than 21 units (4)

End of Block: Section 7 - Dietary Analysis

Start of Block: Section 8 - Additional Information

**Additional Self-reported health and disease characteristics - Optional** During this section, you will be asked questions regarding your health and genetics which we understand can be a sensitive topic for some. If you feel distressed at any point, don't hesitate to get in touch with a research team member so we can provide you with the necessary support. **If you do not know this information, please do not worry and select 'I do not know' or don't answer the questions and click >> to finish the questionnaire. If you don't want to complete this section at all then please scroll down to the bottom of the page and click the >> to finish the questionnaire**. Ellie Miles: e.e.miles@surrey.ac.uk David Bartlett: d.bartlett@surrey.ac.uk

1 What are your most recent complete blood count? If you do not know, leave blank.

- Total White Blood Cell (WBC) count (x109/L) (130) __________________________________________________
- Red Blood Cell (RBC) count (10x9/L) (131) __________________________________________________
- Neutrophil count (132) __________________________________________________
- Lymphocyte count (133) __________________________________________________
- Platelet count (10x9/L) (139) __________________________________________________

2 Cytogenetic testing results, please select all that are relevant to you

- Del[17p] (1)
- Del[11q] (6)
- Del[13p] (7)
- Trisomy 12 (8)
- I do not know (9)
- None (10)

3 Do you have a TP53 mutation?

- Yes (1)
- No (4)
- I do not know (5)

4 Do you have an IGHV (immunoglobulin heavy chain gene) mutation?

- Yes (1)
- No (4)
- I do not know (5)

5 Do you have anaemia?

- Yes (1)
- No (2)
- I do not know (3)

Display This Question:

If Do you have anaemia? = Yes

6 When were you diagnosed with anemia?

________________________________________________________________

Display This Question:

If Do you have anaemia? = Yes

  Are you being treated for anaemia?

- Yes (1)
- No (2)
- I do not know (4)

**You have now finished this questionnaire! To submit the questionnaire please press '>>'.**

End of Block: Section 8 - Additional Information
